# *RNA-Puzzles* Round II: assessment of RNA structure prediction programs applied to three large RNA structures

**DOI:** 10.1261/rna.049502.114

**Published:** 2015-06

**Authors:** Zhichao Miao, Ryszard W. Adamiak, Marc-Frédérick Blanchet, Michal Boniecki, Janusz M. Bujnicki, Shi-Jie Chen, Clarence Cheng, Grzegorz Chojnowski, Fang-Chieh Chou, Pablo Cordero, José Almeida Cruz, Adrian R. Ferré-D'Amaré, Rhiju Das, Feng Ding, Nikolay V. Dokholyan, Stanislaw Dunin-Horkawicz, Wipapat Kladwang, Andrey Krokhotin, Grzegorz Lach, Marcin Magnus, François Major, Thomas H. Mann, Benoît Masquida, Dorota Matelska, Mélanie Meyer, Alla Peselis, Mariusz Popenda, Katarzyna J. Purzycka, Alexander Serganov, Juliusz Stasiewicz, Marta Szachniuk, Arpit Tandon, Siqi Tian, Jian Wang, Yi Xiao, Xiaojun Xu, Jinwei Zhang, Peinan Zhao, Tomasz Zok, Eric Westhof

**Affiliations:** 1 Architecture et Réactivité de l'ARN, Université de Strasbourg, Institut de biologie moléculaire et cellulaire du CNRS, 67000 Strasbourg, France; 2 Department of Structural Chemistry and Biology of Nucleic Acids, Structural Chemistry of Nucleic Acids Laboratory, Institute of Bioorganic Chemistry, Polish Academy of Sciences, 61-704 Poznan, Poland; 3 Institute for Research in Immunology and Cancer (IRIC), Department of Computer Science and Operations Research, Université de Montréal, Montréal, Québec, Canada H3C 3J7; 4 Laboratory of Bioinformatics and Protein Engineering, International Institute of Molecular and Cell Biology in Warsaw, 02-109 Warsaw, Poland; 5 Laboratory of Bioinformatics, Institute of Molecular Biology and Biotechnology, Faculty of Biology, Adam Mickiewicz University, 61-614 Poznan, Poland; 6 Department of Physics and Astronomy, Department of Biochemistry, and Informatics Institute, University of Missouri-Columbia, Columbia, Missouri 65211, USA; 7 Department of Physics, Stanford University, Stanford, California 94305, USA; 8 National Heart, Lung and Blood Institute, Bethesda, Maryland 20892-8012, USA; 9 Department of Physics and Astronomy, College of Engineering and Science, Clemson University, Clemson, South Carolina 29634, USA; 10 Department of Biochemistry and Biophysics, University of North Carolina, School of Medicine, Chapel Hill, North Carolina 27599, USA; 11 Génétique Moléculaire Génomique Microbiologie, Institut de physiologie et de la chimie biologique, 67084 Strasbourg, France; 12 Institut de génétique et de biologie moléculaire et cellulaire, 67400 Strasbourg, France; 13 Department of Biochemistry and Molecular Pharmacology, New York University School of Medicine, New York, New York 10016, USA; 14 Poznan University of Technology, Institute of Computing Science, 60-965 Poznan, Poland; 15 Department of Physics, Huazhong University of Science and Technology, 430074 Wuhan, China

**Keywords:** 3D prediction, bioinformatics, force fields, X-ray structures, models, structure quality

## Abstract

This paper is a report of a second round of RNA-Puzzles, a collective and blind experiment in three-dimensional (3D) RNA structure prediction. Three puzzles, Puzzles 5, 6, and 10, represented sequences of three large RNA structures with limited or no homology with previously solved RNA molecules. A lariat-capping ribozyme, as well as riboswitches complexed to adenosylcobalamin and tRNA, were predicted by seven groups using RNAComposer, ModeRNA/SimRNA, Vfold, Rosetta, DMD, MC-Fold, 3dRNA, and AMBER refinement. Some groups derived models using data from state-of-the-art chemical-mapping methods (SHAPE, DMS, CMCT, and mutate-and-map). The comparisons between the predictions and the three subsequently released crystallographic structures, solved at diffraction resolutions of 2.5–3.2 Å, were carried out automatically using various sets of quality indicators. The comparisons clearly demonstrate the state of present-day de novo prediction abilities as well as the limitations of these state-of-the-art methods. All of the best prediction models have similar topologies to the native structures, which suggests that computational methods for RNA structure prediction can already provide useful structural information for biological problems. However, the prediction accuracy for non-Watson–Crick interactions, key to proper folding of RNAs, is low and some predicted models had high Clash Scores. These two difficulties point to some of the continuing bottlenecks in RNA structure prediction. All submitted models are available for download at http://ahsoka.u-strasbg.fr/rnapuzzles/.

## INTRODUCTION

More than 100,000 structures are currently available in the Protein Data Bank (PDB) (Berman et al. 2000); however, RNA-containing structures take up <6% of these depositions, including RNA structures complexed with other molecules. Although protein related structures constitute >90% of the structure database, <1/1000th of the proteins with known sequences have experimental structures available (Moult 2008). Given the vast number of noncoding RNA molecules being discovered in cells and viruses, it is likely that a very small part of the RNA conformational space has been structurally characterized. RNA structure determination efforts still have a long way to go, and computational modeling could play a major role in providing structural insights for various biological problem explorations.

“RNA-Puzzles” is a CASP-like (Moult et al. 2014) collective blind experiment for the evaluation of three-dimensional (3D) RNA structure prediction. The primary aims of RNA-Puzzles are (i) to determine the capabilities and limitations of current methods of 3D RNA structure prediction based on sequence, (ii) to find whether and how progress has been made, as well as what has yet to be done to achieve better solutions, (iii) to identify whether there are specific bottlenecks that hold back the field, (iv) to promote the available methods and guide potential users in the choice of suitable tools for real-world problems, and (v) to encourage the RNA structure prediction community in their efforts to improve the current tools and to make automated prediction tools available. Until now, 12 puzzles have been set up and assessments of three puzzles were previously published (Cruz et al. 2012).

We now report a second round focusing on the prediction of large RNA structures, a lariat-capping ribozyme (formerly named GIR1), an adenosylcobalamin-binding riboswitch, and a T-box–tRNA complex (Peselis and Serganov 2012; Zhang and Ferre-D'Amare 2013; Meyer et al. 2014). No closely homologous structures existed in structure databases at the time of the experiment, except for the Azoarcus group I intron (Adams et al. 2004) as a potential template for the catalytic core of the GIR1 ribozyme, templates for the tRNA, and crystallographic structure of a segment of a T-box RNA without the tRNA (Wang et al. 2010; Grigg et al. 2013). This round of prediction focuses on (i) the automatic assessment of de novo prediction of large RNA structures, especially structure topology, (ii) the evaluation of the contribution of simple and fast experimental data in structure prediction, such as chemical probing data, and (iii) the identification of bottlenecks in modeling 3D interactions. The ultimate aim is to derive force fields and programming systems allowing for automatic folding of RNA sequences in three-dimensional. However, at this stage, the assessment does not make a distinction between those groups deriving models based solely on ab initio predictions from those incorporating experimental data like chemical probing. As a matter of fact, RNA-Puzzles led to the development of automatic production and retrieval of solution data (see Kladwang et al. 2011a,b, 2014).

For the three puzzles, the best RMSDs range between 6.8 and 11.7 Å, and all display similar topologies to the native structures. Given the sizes of the RNAs (>160 nt), this is a very positive trend for de novo structure modeling. The best models always show much better prediction of non-Watson–Crick interactions but also, surprisingly, relatively high clash scores. This reemphasizes the importance of non-Watson–Crick interactions for RNA 3D structure modeling as well as the difficulty of predicting such interactions on the basis of RNA secondary structure even when complemented with chemical probing data. The observed atomic clashes, possibly due to the inclusions of experimental constraints for nucleotide contacts in the prediction without adequate optimization, have led to further experiments and insight toward better solutions, discussed below.

## THE THREE RNA PUZZLES

### Problem 5: the lariat-capping ribozyme

The lariat-capping ribozyme represents an individual family of ribozymes that has evolved specific architectural features from a group I intron ancestor (Meyer et al. 2014). The LC ribozyme catalyzes a distinct reaction involving formation of a 3-nt 2′,5′ lariat. The 188-nt long sequence is the following:

5′-GGUUGGGUUGGGAAGUAUCAUGGCUAAUCACCAUGAUGCAAUCGGGUUGAACACUUAAUUGGGUUAAAACGGUGGGGGACGAUCCCGUAACAUCCGUCCUAACGGCGACAGACUGCACGGC CCUGCCUCUUAGGUGUGUUCAAUGAACAGU CGUUCCGAAAGGAAGCAUCCGGUAUCCCAAGACAAUC-3′

The crystal structure was resolved to 2.45 Å resolution. Two crystallographic models became available after modeling, with PDB ID's 4P95 and 4P9R.

### Problem 6: the adenosylcobalamin riboswitch

An adenosylcobalamin riboswitch was crystallized (Peselis and Serganov 2012). The 168 nt adenosylcobalamin riboswitch consists of a ligand-bound structured core and a bent peripheral domain. The sequence is the following:

5′-CGGCAGGUGCUCCCGACCCUGCGGUCGGGAGUUAAAAGGGAAGCCGGUGCAAGUCCGGCACGGUCCCGCCACUGUGACGGGGAGUCGCCCCUCGGGAUGUGCCACUGGCCCGAAGGCCGGGAAGGCGGAGGGGCGGCGAGGAUCCGGAGUCAGGAAACCUGCCUGCCG-3′

The crystal structure (PDB 4GXY) has a resolution of 3.05 Å. An adenosylcobalamin molecule is given in the crystal structure but was not revealed at the start of the puzzle.

### Problem 10: the T-box–tRNA complex structure

A T-box–tRNA complex structure was solved (Zhang and Ferré-D'Amaré 2013). The sequence of the 96 nt T-box is as follows:

5′-UGCGAUGAGAAGAAGAGUAUUAAGGAUUUACUAUGAUUAGCGACUCUAGGAUAGUGAAAGCUAGAGGAUAGUAACCUUAAGAAGGCACUUCGAGCA-3′

The sequence of tRNA is the following (75 nt):

5′-GCGGAAGUAGUUCAGUGGUAGAACACCACCUUGCCAAGGUGGGGGUCGCGGGUUCGAAUCCCGUCUUCCGCUCCA-3′

The structure of the complex was solved at a resolution of 3.20 Å (PDB 4LCK). The crystallized sequence was slightly different (the acceptor region was engineered in tRNA), but this detail of the crystal structure was not disclosed in the puzzle. Several RNA modules, including a K-turn, a G-bulge, a double T-loop and an anticodon loop, appeared in this complex structure.

### Additional chemical-mapping data

The Das group provided chemical-mapping data on the three puzzles to all the modelers. One-dimensional chemical-mapping data and mutate-and-map (M^2^) data were acquired, quantitated, and normalized as described in Kladwang et al. (2014) and Seetin et al. (2014), respectively. Three probes were used: 1M7 (a SHAPE reagent, 1-methyl-7-nitroisatoic anhydride, which acylates 2′-hydroxyls of flexible nucleotides); DMS (dimethyl sulfate, reacting with exposed N1/N3 of adenosine/cytosine; and CMCT (1-cyclohexyl(2-morpholinoethyl) carbodiimide metho-*p*-toluene sulfonate, reacting with exposed N1/N3 of guanosine/uracil) ([Mortimer and Weeks 2007; Cordero et al. 2012a] and references therein). Data were released to modelers on the RNA Mapping Database in standardized formats (Rocca-Serra et al. 2011; Cordero et al. 2012b) in accession codes RNAPZ5_STD_0000, RNAPZ5_1M7_0002, RNAPZ5_DMS_0002; RNAPZ6_STD_0001, RNAPZ6_1M7_0002; RNAPZ10_STD_0001, RNAPZ10_STD_0002. Each group was given the possibility to use those data and each group describes below at which stage and how these solution data were used during the modeling process.

## OVERALL COMPARISON RESULTS

### Assessment methods

The automatic model assessment methods were the same as previously used in RNA-Puzzles (Cruz et al. 2012). To geometrically compare predicted models with the experimental structures, we used the Root Mean Square Deviation (RMSD) measure, the Deformation Index (DI), and the complete Deformation Profile matrix (DP) which provides an evaluation of the predictive quality of a model at multiple scales (Parisien et al. 2009). The Clash Score as evaluated by MolProbity is also used as a control measurement for the quality of the geometric parameters of the models (Chen et al. 2010). Additionally, MCQ (Mean of Circular Quantities) score (Zok et al. 2014) was added as a reference to assess prediction in terms of torsion angle space. MCQ measures the dissimilarity between structures taking into account rotatable bonds and sugar pucker. Due to its sensitivity to local differences and independence from structural alignment, it may serve as a complement to methods based on atom coordinates. A single distortion, which can significantly increase global RMSD, influences only distorted residues in case of MCQ. On the other hand, numerous irregularities that sharpen the backbone may cancel out when RMSD is considered, but are revealed in torsion angle space. An implementation of MCQ score is publicly available for download under http://www.cs.put.poznan.pl/tzok/mcq. It allows for several usage scenarios, among which the *global* option was used to assess models in RNA-Puzzles Round II. For each pair consisting of the target and the predicted structure, MCQ-*global* provides a single distance score, representing their mean dissimilarity. Its value was computed upon the differences between the corresponding sugar pseudorotation angle (*P*) and seven dihedral angles defined for a residue (α, β, γ, δ, ε, ζ, and χ). The final rank was built to grasp the overall resemblance of models to the target structure in terms of their trigonometric representation. The MCQ score ranges between 0° and 180°. MCQ-*local* computes raw differences between particular dihedral angles, thus being sensitive even to the smallest discrepancy and allows the observation of high dissimilarity at the residue level. MCQ-*global* introduces an inevitable bias, since the information about single distortions gets lost during the averaging. Therefore an interpretation of the global score should take into account structure size. For large RNAs, *global* MCQ <15° indicates high similarity of structures, while *global* MCQ >45° indicates an overall dissimilarity.

### Problem 5: the lariat-capping ribozyme

**FIGURE 1.**
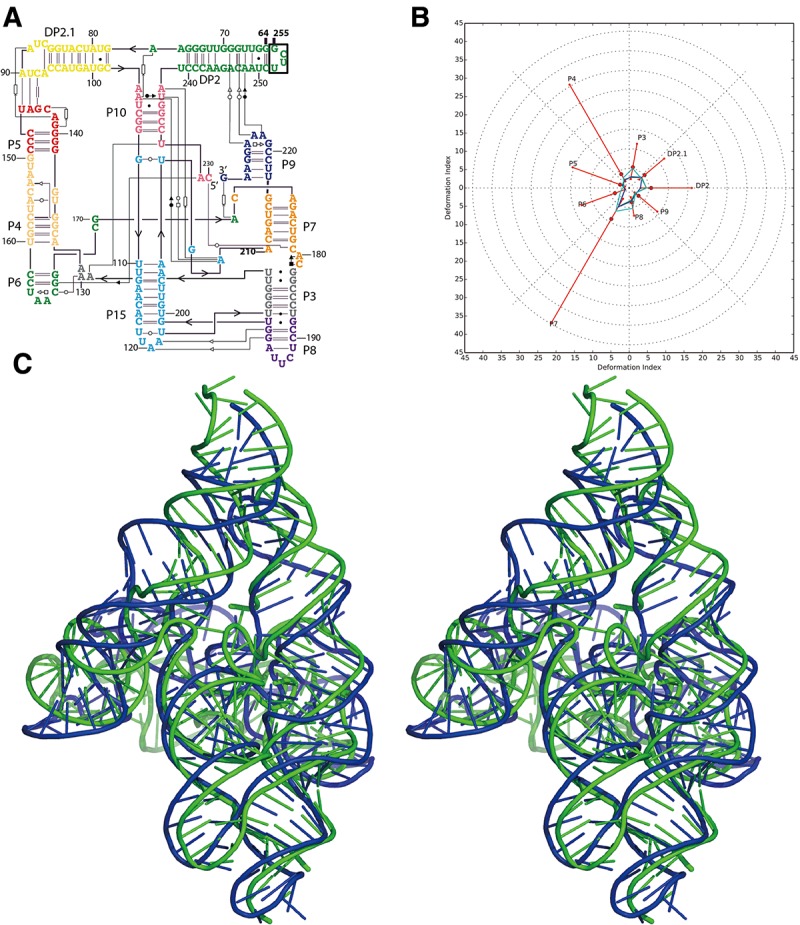
Problem 5: the lariat-capping ribozyme (*A*) secondary structure and (*B*) Deformation Profile values for the three predicted models with lowest RMSD: Das model 2 (green), Das model 1 (blue), and Adamiak model 1 (cyan). (Radial red lines) The minimum, maximum, and mean DP values for each domain. (*C*) Structure superimposition between native structure (green) and best predicted model (blue, Das model 2) with wall–eye stereo representation.

**FIGURE 2.**
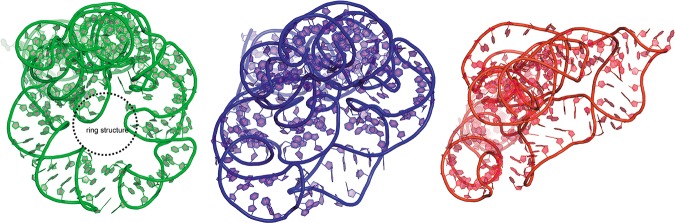
Illustration of the “ring” topology structure in Problem 5. Native structure with “ring” topology is shown in green; the best prediction model Das model 2 and the third best prediction Adamiak model 1 are shown in the same aspect in blue and red, respectively. Although the best model cannot totally capture the “ring” topology, it is more similar to native topology than others.

A total of 25 predicted models were submitted with RMSDs ranging from 9.15 to 36.5 Å (mean RMSD is 24.1 Å, see Table 1). The top two models are better than the others in terms of RMSD, Deformation Index, non-Watson–Crick interactions and stacking (Fig. 1). The top three models also have >90% Watson–Crick (WC) base pairs correctly predicted. Most of the groups have, however, submitted models with very low accuracy in non-WC interactions. The last three models ranked by RMSD have similarly poor levels of accuracy for non-WC interactions, with worse prediction of WC pairs as well as worse stacking predictions. Several models present high values for the Clash Score (Chen et al. 2010), while the Clash Score for the crystal structure is low at 5.86. This implies a need for updated dictionaries of distances and angles or stronger constraints toward reasonable values both during crystal structure refinement and in structure modeling. The crystal structure of problem 5 shows an open ring structure around the center formed by a kissing interaction between two peripheral helices. This striking architecture with a clearly visible “hole” through the ring is not exactly predicted by any of the prediction models, although some groups correctly identified the overall topology (Fig. 2).

**TABLE 1.**
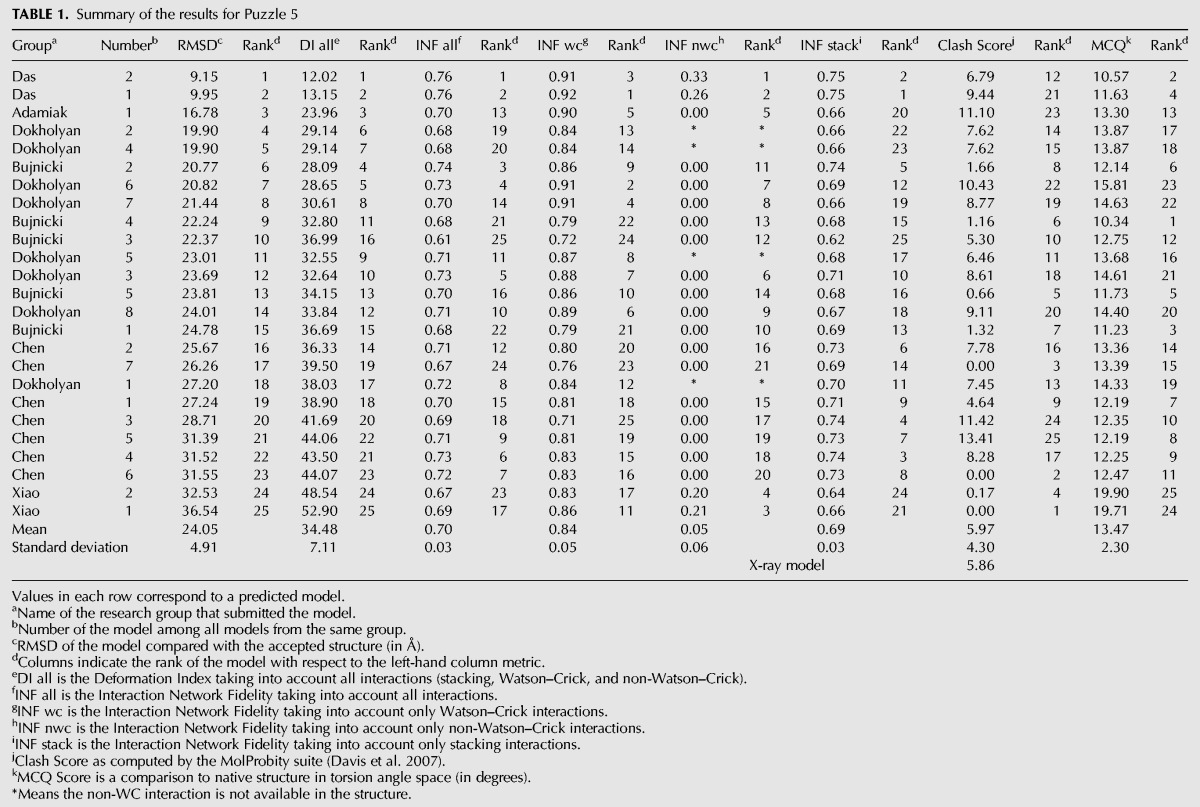
Summary of the results for Puzzle 5

### Problem 6: the adenosylcobalamin riboswitch

The RMSDs of the 34 submitted prediction models range from 11.4 to 37.0 Å with a mean value of 23.1 Å (Table 2). These are very large RMSDs but the noninclusion of the ligand in the puzzle could be largely the cause of such high values. The Das, Major, and Chen groups rank at the top as they have relatively high accuracies in non-WC interaction prediction, while the other groups do not have the correct non-WC interactions. As a large riboswitch structure, the native structure has a Clash Score of 7.98. In such a situation, it is probably understandable that clashes appear in prediction models in order to maintain the same topology as the native structure. Models from the Das group show much better similarity to the native structure but with much higher Clash Scores due to the limited time available for refining models for this target.

**TABLE 2.**
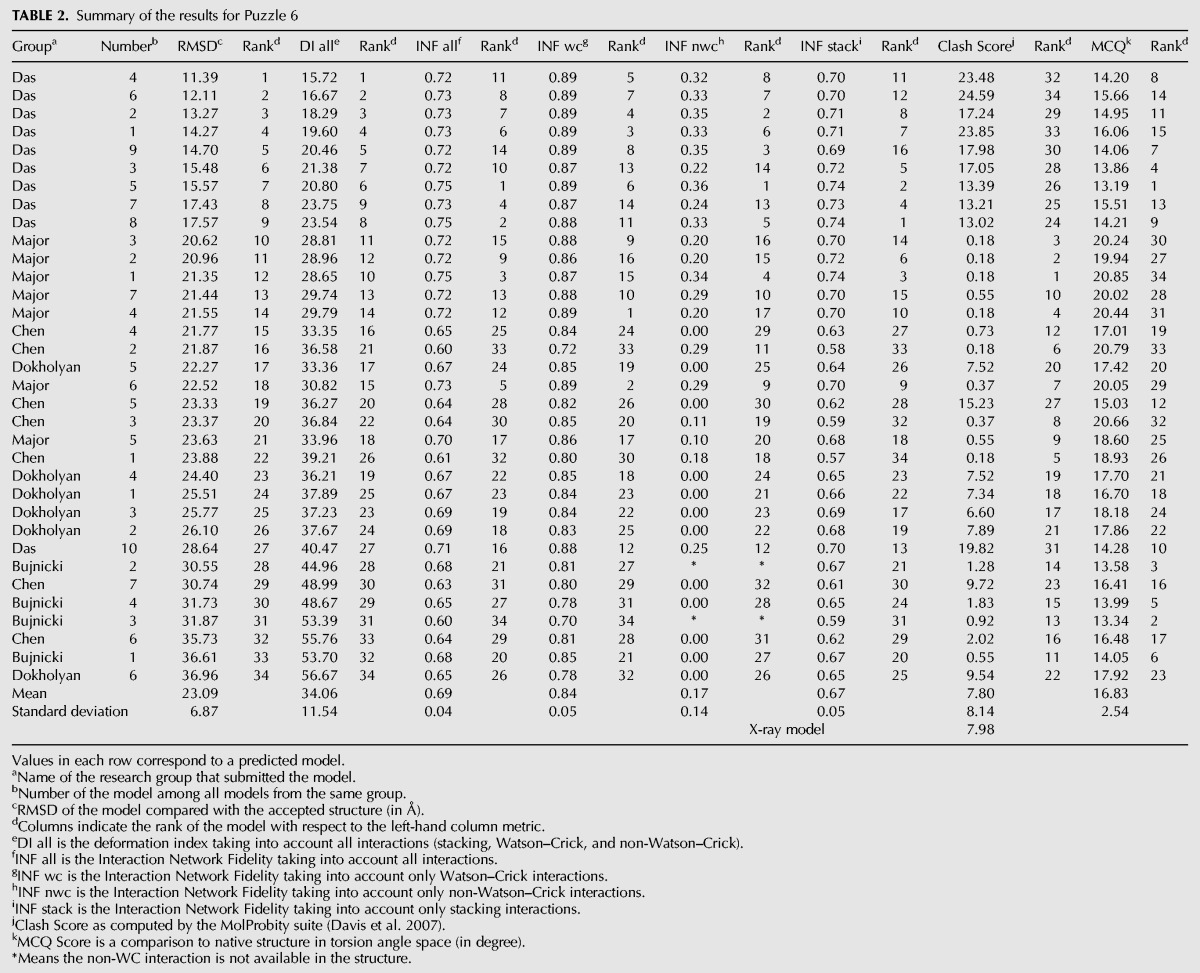
Summary of the results for Puzzle 6

### Problem 10: T-box–tRNA complex

Twenty-six prediction models were submitted ranging from 6.8 to 16.9 Å RMSD with 11.5 Å as the mean value (Table 3). As this is a complex of two RNA molecules, we also compared the models of each molecule separately. The RMSDs of the T-box ranges from 5.96 to 17.9 Å, with a mean RMSD of 12.1 Å, exactly the same as the average RMSD of the molecular complex. As the structure topology of tRNA is well known, the modeling is more accurate, and the average RMSD achieved is 3.8 Å with a RMSD range between 2.49 and 6.9 Å. Therefore, the key comparisons between predictions are the T-box structure and the relative orientation/interaction between the T-box and the tRNA. We find models of the tRNA segment from the Bujnicki group rank at the top, while the Das group does better for both the T-box alone and overall T-box/tRNA models. For the T-box, the Das group shows good predictions for both WC base pairs and non-WC interactions. However, their models, even the tRNA structures, involve more atomic clashes. In comparison, the Bujnicki group achieved better accuracy on tRNA and included fewer atomic clashes.

**TABLE 3.**
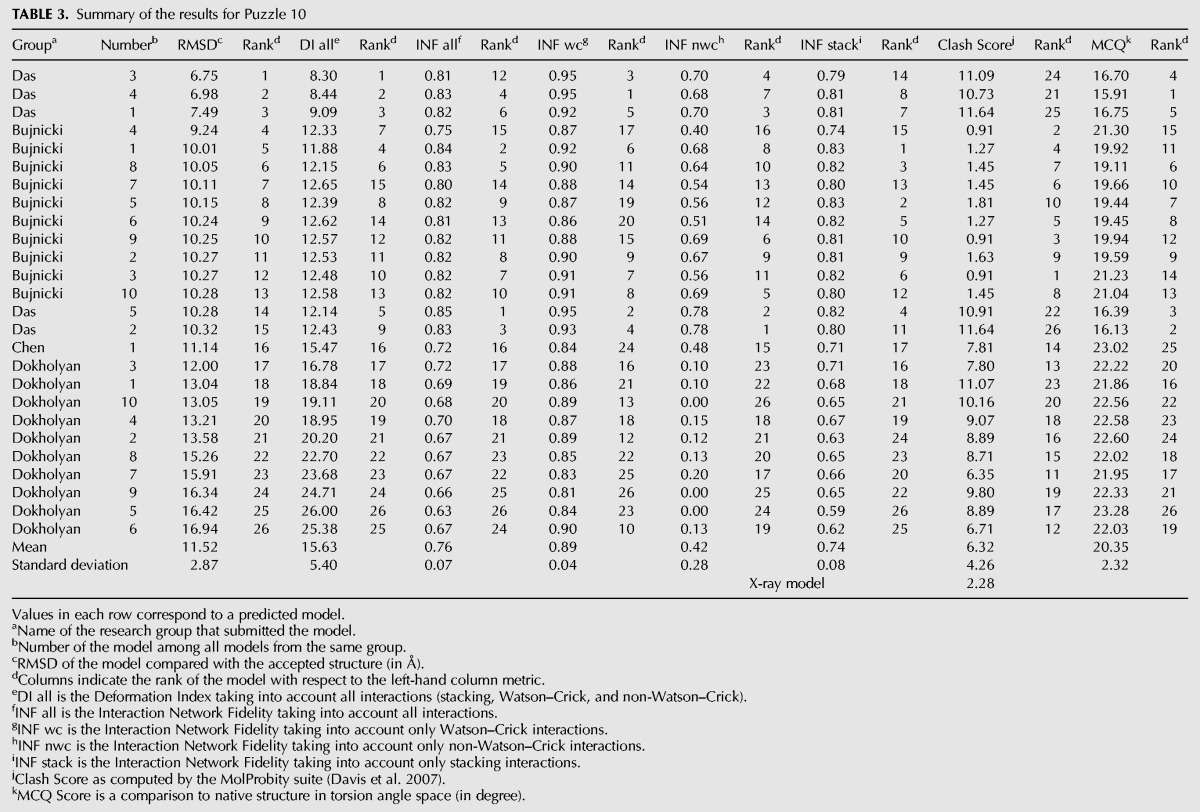
Summary of the results for Puzzle 10

## MODELING METHODS

Seven research groups pursuing the development of automatic modeling approaches participated in this round of RNA-Puzzles experiments. The following provides a brief description of the methodology and protocols used by the modeling groups (arranged alphabetically), together with comments and discussions.

### Adamiak group

The RNA 3D structure from the Adamiak group was predicted using automated method RNAComposer (Popenda et al. 2012) in its batch mode. RNAComposer server (http://rnacomposer.cs.put.poznan.pl) uses sequence and secondary structure topology information in dot-bracket notation. Secondary structure (using the RNAStructure software) (Reuter and Mathews 2010) was adjusted using experimental data given for that RNA sequence by the Das group. Additional information about potential pseudoknots or tertiary contacts was obtained from manual analysis of the mutate-and-map data provided by the Das laboratory (RNA Mapping Database).

For Problem 5, two interactions were found: (i) 28UC29 with 93GA94 and (ii) 111GACUG115 with 148CAGUC152. Both were introduced as squared brackets into extended dot-bracket notation input:

GGUUGGGUUGGGAAGUAUCAUGGCUAAUCACCAUGAUGCAAUCGGGUUGAACACUUAAUUGGGUUAAAACGGUGGGGGACGAUCCCGUAACAUCCGUCCUAACGGCGACAGACUGCACGGCCCUGCCUCUUAGGUGUGUUCAAUGAACAGUCGUUCCGAAAGGAAGCAUCCGGUAUCCCAAGACAAUC
(((((..(((((..(((((((((....[[.)))))))))..(((((((((((((......((((....((((((((((..]]))))....)).))))((....)).....[[[[[....)))).(((.....)))))))))).....]]]]].((((....))))..))))))...)))))..)))))

Before pressing “Compose” button in the batch mode, the option “Add atom distance restraints” was checked to introduce restraints concerning the interactions 111GACUG115 with 148CAGUC152. To do so, the RNA duplex with the same sequence was extracted from the X-ray structure (PDB 2Z75, resolution 1.7 Å) and selected using search engine of RNA FRABASE (Popenda et al. 2008). Subsequently, related 508 distance restraints were calculated (between atoms P, C1′, C2′, C5′, O3′, C2, C4, C6, and C8) and uploaded to RNAComposer. RNAComposer (64*-*bit Intel Xeon 2.33 GHz processor-based platform with scalable 8 GB memory) predicts 10 3D models within <10 min.

The resulting models were inspected for the total energy value calculated by RNAComposer and for the preservation of the 111GACUG115/148CAGUC152 and 28UC29/93GA94 interactions. Models showing lowest total energy were chosen for further analysis. Some fragment selections chosen by RNAComposer for the 3D structure assembly prohibited the formation of required contacts and such models were rejected at this stage. Subsequently, the selected models were investigated for the proximity of the 111GACUG115/148CAGUC152 pseudoknot region to the RNA termini. The mutate-and-map data (RNA Mapping Database) suggested that region hosting pseudoknot 111GACUG115/148CAGUC152 should be close to the molecule termini, namely to the internal loop 6GG7/182GA183. The model fulfilling this criterion and representing the lowest total energy estimated by RNAComposer was selected. Since RNAComposer automatically conducts two energy minimization steps prior to returning final RNA 3D structure this model did not require any further refinement. The model was validated using NUCheck (Feng et al. 1998).

### Bujnicki group

The Bujnicki group used a hybrid strategy similar to the one used in the previous editions of the RNA-Puzzles experiment (Cruz et al. 2012), which comprised template-based (comparative) modeling, global folding with restraints using a coarse-grained method for template-free folding, and high-resolution refinement.

First, for all target sequences they attempted to identify homologous families in the Rfam database (Burge et al. 2013) and homologous RNAs with experimentally determined structures. For RNA sequences or sequence fragments that exhibited homology with RNAs with experimentally determined structures, initial models were constructed by template-based modeling and fragment assembly using ModeRNA (Rother et al. 2011). Target-template alignments were prepared manually, with the aid of secondary structure information extracted from Rfam and corrected if needed with the use of predictions made with RNA metaserver (http://genesilico.pl/rnametaserver/) developed as a part of the CompaRNA project (Puton et al. 2013). This stage was very similar to that which they used previously in RNA-Puzzles (Cruz et al. 2012). For Problem 5, a lariat-capping ribozyme related to group I self-cleaving introns (Rfam family RF01807), they used a group I intron structure (PDB 1ZZN) as the main template. For Problem 6, an adenosylcobalamin riboswitch (RF00174), they were unable to find a suitable template, therefore no template-based modeling was performed. For Problem 10, a tRNA bound to a T-box RNA, template-based modeling of the entire complex was based on the structure of a related complex (Grigg et al. 2013) built manually by the authors based on crystal and NMR structures of fragments (PDB 4JRC and PDB 2KHY), with the use of cross-linking, mutagenesis, and SAXS data.

The aforementioned initial models of target structures (in the case of Problem 6—an artificial circular conformation of the target sequence with 5′ and 3′ ends close to each other) were used as starting points for global refinement, using the SimRNA method for RNA folding simulations, which uses a coarse-grained representation, relies on the Monte Carlo method for sampling the conformational space, and uses a statistical potential to approximate the energy and identify conformations that correspond to biologically relevant structures (MJ Boniecki, G Lach, K Tomala, W Dawson, P Lukasz, T Soltysinski, KM Rother, and JM Bujnicki, in prep.). Here, they used a novel version of SimRNA, which uses five (rather than three) atoms per residue: P of the phosphate group, C4′ of the ribose moiety, and in which base moieties are represented by triangles: N1–C2–C4 for pyrimidines and N9–C2–C6 for purines. This representation provides much improved description of base faces and edges compared with the previous version that used only one atom per base (Cruz et al. 2012; Rother et al. 2012) and therefore improves the modeling of stacking and base-pairing interactions, e.g., it discriminates much better between canonical and noncanonical base-pairing. Regions predicted to be confidently modeled in initial models were “frozen” while other regions were allowed to change conformation. For modeling of complex 3D structures, SimRNA can use additional restraints, derived from experimental or computational analyses, including information about secondary structure and/or long-range contacts. They have used such information depending on its availability. Typically, predictions were first made with restraints on predicted secondary structure and if additional data became available sufficiently long before the prediction deadline (e.g., results of experiments performed by the Das group and made available to all participants of RNA-Puzzles), additional simulations were conducted. Given the very tight deadline for Problem 6, they were unable to utilize additional data for this RNA, leading to poor results.

Predictions generated by SimRNA were converted to full-atom representation and ranked for submission using a combination of various criteria, including the results of clustering (the higher number of similar well-scored structures the better), agreement with experimental data not used in the process of modeling, manual inspection, and scoring with independent methods such as RASP (Capriotti et al. 2011). If time permitted, models selected for submission were subjected to high-resolution refinement whose aim was to reduce clashes, idealize geometries, and improve local interactions such as in standard and non-WC base pairs. Here, they used a different method than previously, namely an in-house software tool QRNAS (J Stasiewicz and JM Bujnicki, unpubl.) that extends the AMBER force field with energy terms explicitly modeling hydrogen bonds, idealizes base pair planarity and regularizes backbone conformation.

As in their previous (Cruz et al. 2012) modeling exercise, human intervention was relatively large. Most of the time was devoted to searching for additional information related to target RNA sequences and discussions within the group. Time used for alignment preparation and for selection of models for submission varied greatly depending on the difficulty of the Problem. Time used for template-based modeling was negligible. Time required for SimRNA modeling was typically a few days per target, and the final refinement was typically run overnight.

### Chen group

The Chen group used a hierarchical approach to predict RNA 3D structure from the sequence (Xu et al. 2014). For a given RNA sequence, they first predict the secondary structure from the free energy landscape using the Vfold model (Cao and Chen 2005, 2006, 2009; Chen 2008). A unique feature of the Vfold model at secondary level is its ability to compute the RNA motif-based loop entropies. Using two virtual bonds per nucleotide to represent the backbone conformation, Vfold model samples fluctuations of loops/junction conformations in 3D space through conformational enumeration model (Cao and Chen 2005, 2006, 2009; Chen 2008). By calculating the probability of loop formation, the model can give the conformational entropy parameters for the formation of the different types of loops such as pseudoknot loops. Another notable feature of the Vfold model at secondary level is the modeling of RNA loop free energy. By enumerating all the possible (sequence-dependent) intra-loop mismatches, the Vfold model partially accounts for the sequence-dependence of the loop free energy.

Next, a 3D coarse-grained scaffold is constructed based on the predicted secondary structure (Cao and Chen 2011). To construct a 3D scaffold, the predicted helix stems are modeled as A-form helices. For the loops/junctions, 3D fragments from the known PDB database were used. Specifically, a structural template database (Xu et al. 2014) was built by classifying the structures according to the different motifs such as hairpin loops and internal/bulge loops, three-way junctions, four-way junctions, pseudoknots, etc. For each junction, the optimal (top 5) fragments were selected for the further structure assembly of the whole RNA. Any 3D structures generated by the structure assembly with structural clashes would be excluded. The final all-atom structures were built based on the coarse-grained model, followed by refinement using AMBER energy minimization. Two thousand steps of energy minimization were run, applying 500.0 kcal/mol constraints to all the residues, followed by another 2000 steps of minimization without constraints.

In order to increase the accuracy of RNA secondary structure prediction, they applied Rfam (Burge et al. 2013) to identify the possible conserved base pairs and used the most conserved base pair information as constraint to the Vfold algorithm to predict secondary structures. If available, the SHAPE (selective 2′-hydroxyl acylation analyzed by primer extension) (Merino et al. 2005) experimental data were also used as constraint in the Vfold algorithm for secondary structure prediction. The SHAPE reactivity is strongly related to the nucleotide flexibility at single nucleotide resolution. Specifically, some nucleotides are restricted to be in loop regions (without forming base pairs with other nucleotides) because of their high SHAPE reactivity. The combination of SHAPE data and/or homologous sequence information from Rfam and the Vfold algorithm led to enhanced accuracy of RNA secondary structure prediction.

For the RNA/RNA complex of Problem 10, they built the 3D structures for each strand separately using the above hierarchical approach (Xu et al. 2014). The final complex structure was built manually based on the previously published SAXS-reconstructed envelope from DAMMIF (Fig. 5 in Grigg et al. 2013). Then, they ran a short-time MD simulation to stabilize the interactions between the two RNA molecules.

In summary, the computation involved two steps: (a) the prediction of the secondary structure and the construction of the coarse-grained 3D structure and (b) AMBER energy minimization. The computation time (Ta, Tb) for the two steps are (∼2–3 h, <1 h), (<1 h, <1 h), and (<1 h, <3 h), for Problems 5, 6, and 10, respectively. The computations were performed on a desktop PC with Intel Core(TM) i7-2600 CPU at 3.40 GHz. They manually incorporated the constraints from the SHAPE experiments and the Rfam results into the Vfold model for secondary structure prediction. In addition to the construction of 3D structures, the RNA/RNA complex for Problem 10 involved human interference based on the SAXS data. All other steps were achieved automatically by computations.

### Das group

**FIGURE 3.**
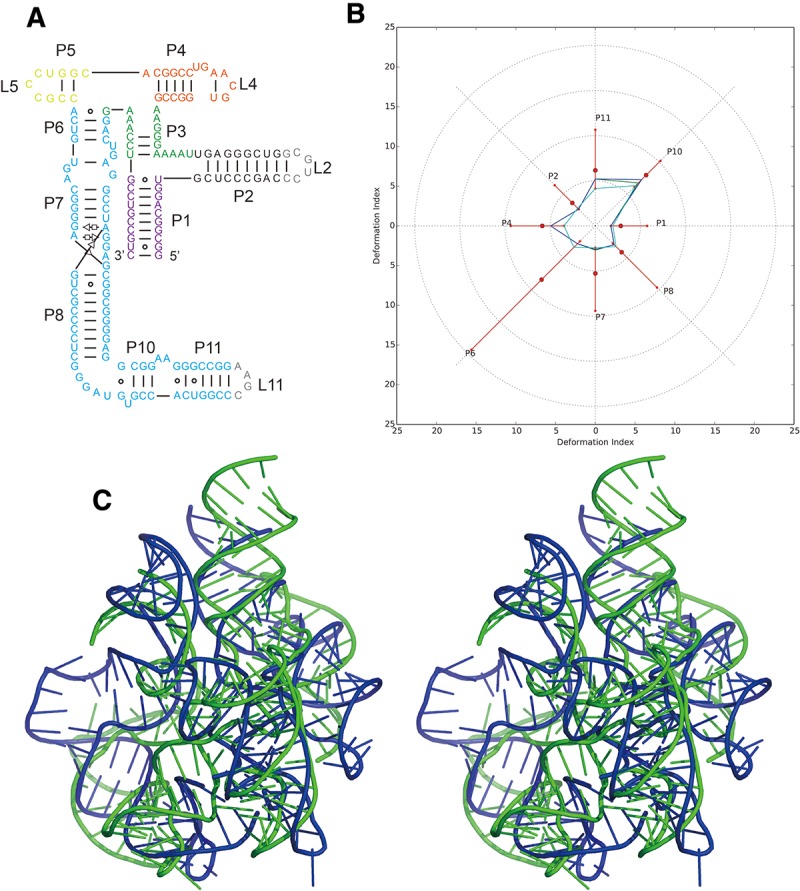
Problem 6: the adenosylcobalamin riboswitch (*A*) secondary structure and (*B*) Deformation Profile values for the three predicted models with lowest RMSD: Das model 4 (green), Das model 6 (blue) and Das model 2 (cyan). (Radial red lines) The minimum, maximum, and mean DP values for each domain. (*C*) Structure superimposition between native structure (green) and best predicted model (blue, Das model 4) with wall–eye stereo representation.

The chemical probing data, obtained in by the Das group, were first used to model secondary structure with available automated algorithms and then further used for tertiary structure prediction. Automated secondary structure modeling with RNAstructure was carried out as described in Tian et al. (2014), with tools available by server (http://rmdb.stanford.edu/structureserver/) (Cordero et al. 2012b). In all three Problems, use of 1D SHAPE data improved RNAstructure 5.4 secondary structure predictions compared with modeling without data, leading to perfect recovery of helices in Problem 10 (Supplemental Table S1; Supplemental Fig. S6B). Nevertheless, approximately half of helices remained mispredicted in Problems 5 and 6. In Problem 5, use of DMS/CMCT with RNAstructure gave better models than SHAPE-guided modeling; but for Problems 6 and 10, use of DMS/CMCT made models worse compared even with modeling without data. On an encouraging note, a bootstrapping procedure that gives conservative estimates of modeling uncertainty (Kladwang et al. 2011b; Ramachandran et al. 2013) was able to highlight confident and nonconfident regions in all cases. For example, any helix modeled with >75% bootstrap values agreed with the subsequently released crystallographic model (Supplemental Figs. S1–S3) (Fig. 3).

**FIGURE 4.**
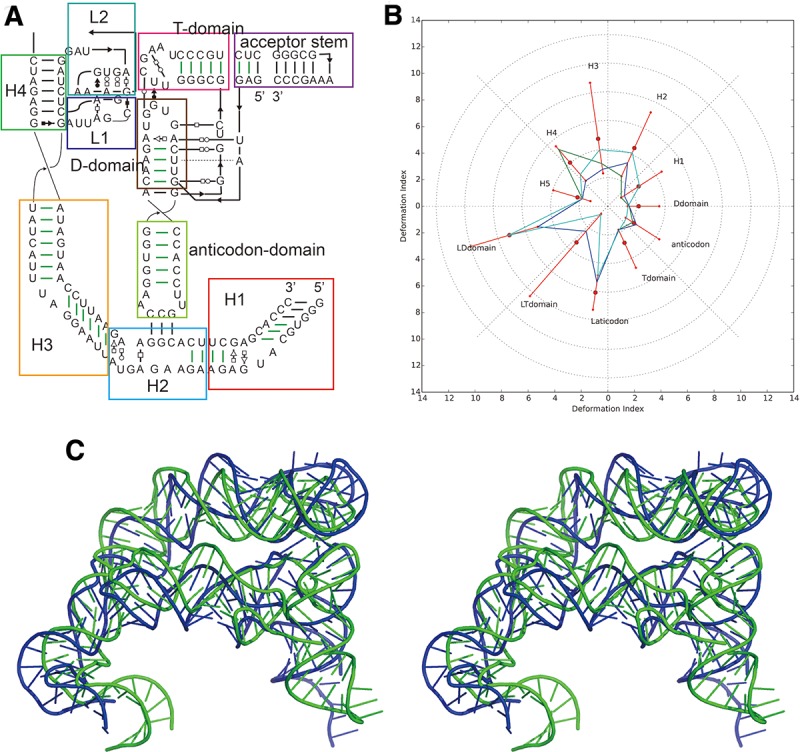
Problem10: the T-box–tRNA complex (*A*) secondary structure and (*B*) Deformation Profile values for the three predicted models with lowest RMSD: Das model 3 (green), Das model 4 (blue), and Das model 1 (cyan). (Radial red lines) The minimum, maximum, and mean DP values for each domain. (*C*) Structure superimposition between native structure (green) and best predicted model (blue, Das model 3).

**FIGURE 5.**
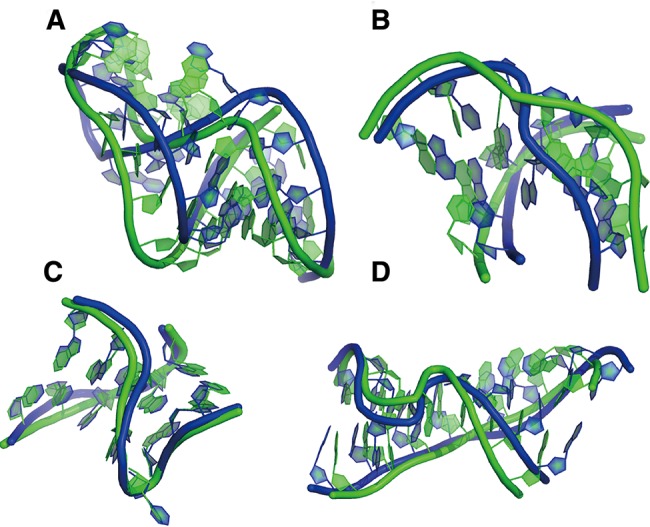
Modules in Problem 10. (*A*) Detailed structure of T-loop of Das model 4, (*B*) detailed structure of U30 of Das model 4, (*C*) detailed structure of K-turn of Das model 4, (*D*) detailed structure of Loop-E of Das model 4.

More recent versions of RNAstructure have updated parameters for nearest-neighbor energies and for converting SHAPE values to pseudoenergies, and also have the ability to model pseudoknots (Hajdin et al. 2013). Although not a strictly blind test, the data above allowed a test of these advances. Use of RNAstructure 5.6 *Fold* for SHAPE-directed modeling did not improve modeling of Problem 5, and gave less accurate models of the other Problems 6 and 10, compared with RNAstructure 5.4 *Fold*; the difference appears to be due to a change in the parameters for SHAPE pseudoenergy. Use of the *ShapeKnots* executable produced a significant improvement but still imperfect model in SHAPE-directed modeling of Problem 5, which contains a pseudoknot in its catalytic core. In the other cases, *ShapeKnots* predictions did not improve upon pseudoknot-free *Fold* modeling. These results underscore the challenge of modeling RNA secondary structure using conventional 1D chemical-mapping data, even with continuing algorithmic advances. As has been discussed previously (Cordero et al. 2012a; Leonard et al. 2013; Rice et al. 2014), protection of nucleotides may signal non-Watson–Crick rather than Watson–Crick pairing in the structure, but current methods do not generally distinguish these possibilities (Figs. 4, 5).

For Problems 5 and 6, secondary chemical-mapping data were also acquired through the M^2^ approach (Cordero et al. 2014). In this method, chemical-mapping profiles are measured not only for the sequence of interest but also for variants mutating each nucleotide in the RNA (Kladwang et al. 2011a). Increased reactivity of one nucleotide upon mutation of a sequence-distance nucleotide can signal their interaction in three dimensions, and these data can be leveraged for automatic secondary structure inference in RNAstructure. For Problem 5, RNAstructure 5.4 *Fold* guided by M^2^-SHAPE data recovered all helices longer than 2 bp, except the catalytic core pseudoknot. Integrating M^2^ data with the more recent RNAstructure 5.6 *ShapeKnots* recovered all of these helices, including the pseudoknot (Supplemental Table S1; Supplemental Fig. S1L). For Problem 6, all helices longer than 2 bp were recovered correctly with M^2^-SHAPE data and by either RNAstructure executable. Errors in edge base pairs of several helices remain, as well as a register shift in Problem 5 (which was corrected in M^2^-DMS analysis; Supplemental Fig. S1P). Overall, these comparisons confirm that secondary chemical mapping coupled to automated algorithms consistently achieves correct global secondary structures for complex RNA folds in terms of helix recovery, but resolving fine errors in edge base pairs will require methodological improvements.

Beyond basic secondary structure modeling, this round of puzzles also inspired development of computational methods for 3D modeling, primarily in three areas. First, simple automated tools in the Rosetta framework (Leaver-Fay et al. 2011) were created for threading structural templates into the desired sequence, such as the catalytic core of Problem 5, the lariat-capping ribozyme (see also below). Second, the Das group expanded fragment assembly of RNA with full-atom refinement (FARFAR) (Das et al. 2010; Kladwang et al. 2011a), whose interface for job setup was previously cumbersome, especially to solve subpieces of large RNAs (e.g., the three-way P15/P8/P3 junction of Problem 5). The RNA puzzles inspired them to write a single python script (rna_denovo_setup.py) for straightforward setup of FARFAR jobs, taking as input the full-model sequence and secondary structure, the residues of desired subdomain, and PDB models for any known subpieces of the subdomain. For Problem 6, the Das group also created a mode for setting up rigid-body “docking” of multiple RNA pieces including a placeholder sphere for the adenosylcobalamin ligand. Finally, an expansion of “stepwise” assembly was piloted, previously developed for enumerative high-resolution modeling of motifs (Sripakdeevong et al. 2011; Chou et al. 2013), to gene rate complex RNA folds by progressively closing “rings” of motifs through numerous tertiary buildup paths (e.g., to model the ring-like connection of the catalytic core with the P3/P8/P15 junction and the P2.1/P5 kissing loops in the GIR1 lariat-capping ribozyme). Compared with prior fragment assembly approaches from Das group (Kladwang et al. 2011a), this stepwise strategy was efficient in generating realistic, converged conformations of complex folds with multiple tertiary contacts at subhelical resolution. All Rosetta tools are freely available to academic researchers (Leaver-Fay et al. 2011) and documented at https://www.rosettacommons.org/docs/latest/rna-denovo-setup.html/.

The process was a mix of automatic and manual steps, as many of the tools were being developed “on-the-fly.” On one hand, chemical-mapping-guided inference of secondary structure to double-check models from the literature was carried out automatically, but inference of some tertiary contacts from these data was guided by visual inspection of the M^2^ data (see below). On the other hand, identification of potential templates for threading/homology modeling was not carried out automatically. Structural templates and alignments were instead derived from literature search (group I intron alignment to the lariat-capping ribozyme for Problem 5 (Beckert et al. 2008); mapping “half” of the FMN riboswitch to the adenosylcobalamin-binding core for Problem 6 (Barrick and Breaker 2007; Geary et al. 2011); and mapping double T-loop (Grigg et al. 2013; Lehmann et al. 2013), sarcin–ricin loop (Yang et al. 2001) and kink-turn motifs (Vidovic et al. 2000) to T-box, the tRNA (Fukai et al. 2000), and the T-Box/tRNA interface derived from ribosome (Dunkle et al. 2011) for Problem 10) and manual expert inspection (refining the core of the Problem 5 group I intron alignment; a previously unrecognized kink-turn in the Problem 6 adenosylcobalamin riboswitch; the ribosome-bound tRNA/mRNA-like interaction in the Problem 10 T-box/tRNA complex). Fully automating template recognition and tertiary contact inference-with guidance from readily available chemical-mapping data-appears to be an important challenge for the field (Table 4).

**TABLE 4.**
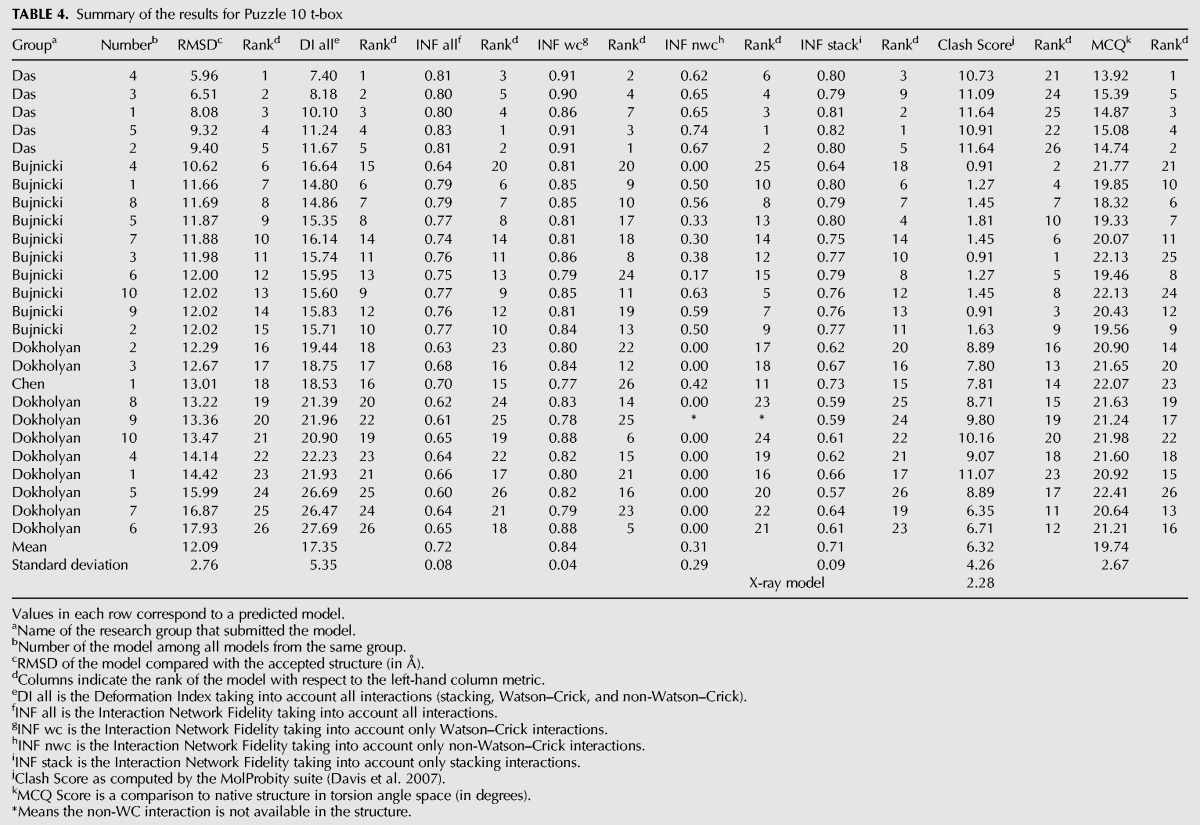
Summary of the results for Puzzle 10 t-box

For all three targets, experimental data were critical for ruling out structural hypotheses that would have required substantial computational expense to explore, and in some cases, gave critical data that guided modeling, illustrated in Supplemental Figures S4–S6. For Problem 5, two peripheral tertiary contacts were not recognized in previous literature but were important for defining ∼1/3 of the model. The contacts were apparent in M^2^ data as changes in chemical mapping on one side of the contact in response to mutations on the other side. For Problem 6, several secondary structure models had been proposed in the literature (Ravnum and Andersson 2001; Nahvi et al. 2002, 2004; Vitreschak et al. 2003; Barrick and Breaker 2007), and the M^2^ analysis was important for unambiguously confirming the correct model. Further, the M^2^ data showed no evidence of extensive interaction between the helix P2 of the P1/P2/P3 junction and the long “arm” P7–P11, or for interactions within the “arm”; so runs with those contacts were not set up. For Problem 10, M^2^ data was not acquired due to time constraints (three other Problems were being modeled concomitantly), but the available 1D chemical-mapping data helped rule out a potential fourth base pair neighboring the three base pair interaction of the tRNA anticodon and its T-box binding site; enforcing that interaction would have produced inaccurate distortions (Table 5).

**TABLE 5.**
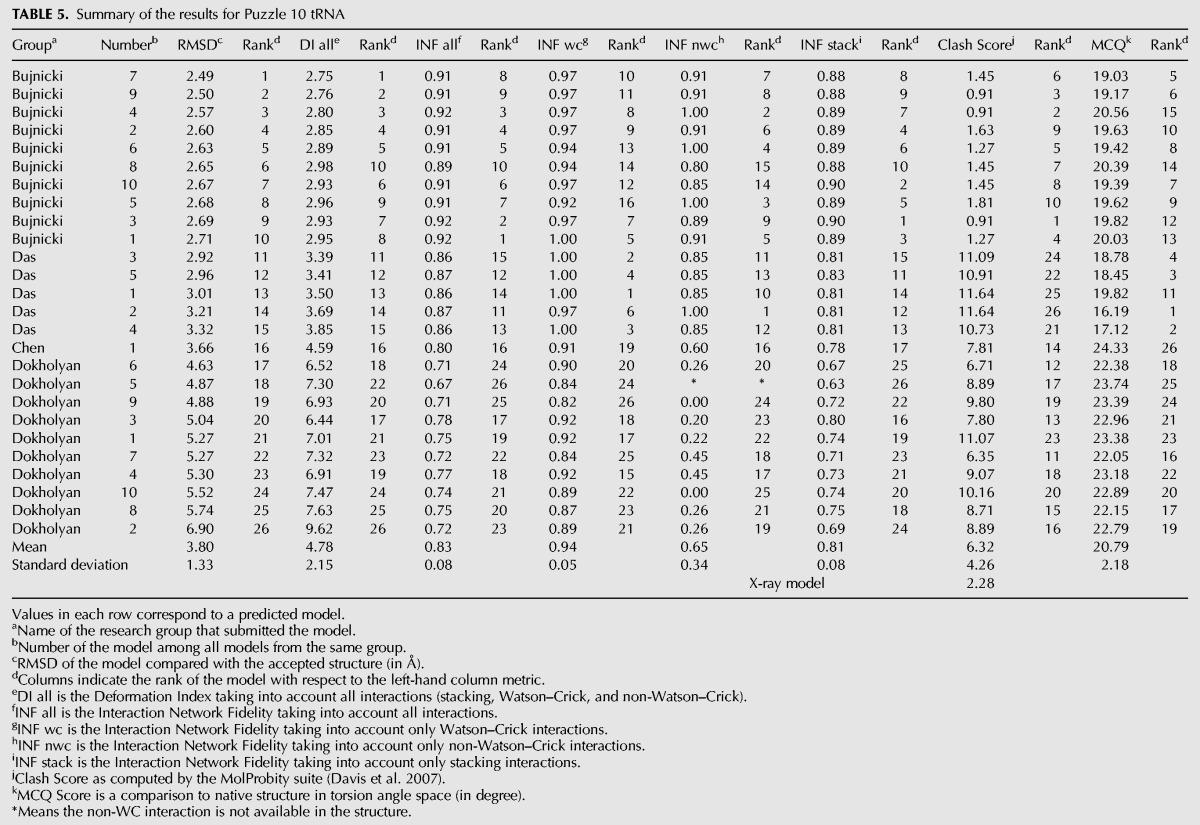
Summary of the results for Puzzle 10 tRNA

As many of the computational methods were being developed at the same time as modeling, performance was not optimized. Thousands of CPU-hours were used (12 h for ∼20–100 cores) for each 3D modeling step that involved fragment-based assembly and refinement of subpieces. For the case of Problem 5, the Das group ended up expending at least 30,000 CPU-hours. Nevertheless, since the prediction period, further automation and optimization has brought the computational expense of these procedures to under 10,000 CPU-hours per target, taking less than a week of wall clock time. It is noted that academic researchers interested in using these tools can make use of free “startup” allocations on the XSEDE supercomputers of up to 20,000 CPU-hours. As for Problem 6 (adenosylcobalamin riboswitch), both experiments and computational modeling were carried out in 1 wk.

### Dokholyan group

The Dokholyan group at the University of North Carolina at Chapel Hill in collaboration with the Ding group from Clemson provided predictions for Problems 5, 6, and 10 using multiscale discrete molecular dynamics (DMD) method (Proctor et al. 2011; Shirvanyants et al. 2012). The structure modeling was performed with coarse-grained folding simulations followed by an all-atom reconstruction. In the coarse-grained folding simulations, the three-bead RNA model, where each nucleotide is represented by three pseudoatoms corresponding to base, sugar, and phosphate groups (Ding et al. 2008), was used. The interactions between the three beads are modeled based on information available from high-resolution RNA structure database. Bonded interactions are based on parameters derived from covalent-bonding, bond angles and dihedral angles, while the nonbonded interactions are derived from base-pairing, base stacking, hydrophobic interactions, and phosphate–phosphate repulsions. Replica exchange DMD simulations were performed (Ding et al. 2008) followed by a selection protocol to select the lowest energy structures. Briefly, structures were selected from coarse-grained simulations based on energies obtained using the coarse-grained energy function. In the first filter, all the structures from every replica, which are the lowest ten percent of the energies and then perform hierarchical clustering for identifying the most dominant state among the lowest energy ensemble, were selected. The centroid of the most populated cluster was selected as the representative structure for the simulation. For the representative structure, the model was further refined by performing all-atom reconstruction. The all-atom DMD approach for RNA is similar to one used for all-atom protein modeling (Ding et al. 2008).

The CPU time for DMD-based RNA structure prediction depends on the length of the RNA. Previous benchmarks’ showed linear dependence on RNA length. For Problems 6 and 10, the simulations were performed on UNC Killdevil computing cluster (each compute node consists of 12 core, 2.99 GHz Intel processors, with either 48 of 96 Gb memory), for Problem 6, a 169-nt length RNA, the total CPU time was ∼21 h for eight compute nodes, which roughly translates to ∼2.75 h of real time in simulation. The clustering algorithm run on similar compute node took <15 min to complete.

In the predictions they used base-pairing information, which was derived for each Problem using different methods. Problem 5 was mainly based on biochemical data provided by the Das group and sequence comparative analysis obtained by multiple sequence alignment from Rfam (Griffiths-Jones et al. 2003). The same was true for Problem 6 with additional data obtained from the Mfold server (Zuker 2003). Problem 10 used biochemical data from the Das group and the Mfold server (Zuker 2003).

Experimentally derived tertiary structure information was also used. In Puzzle 5, the specific long-range proximity constraint between nucleotides 78 and 170 was inferred from the cleavage sites between helices 5 (P5) and 10 (P10). For Problem 6, the two groups used the in-line probing data to approximate the solvent accessibility (Nahvi et al. 2004), which were added to DMD simulation as the hydroxyl radical probing approach (Ding et al. 2012). In the case of Problem 10, the two binding sites between tRNA and tBox were taken from experimental study (Grigg et al. 2013). The method is fully automated once the base-pairing information for the RNA has been provided.

### Major group

The adenosylcobalamin riboswitch of Problem 6 was found by sequence similarity using BLAST. The secondary structure for this riboswitch was deduced by Barrick and Breaker (2007) and it was used as a primary template. Among the alternative structural elements, they kept the three-way and four-way junctions, as well as the T-loop-type interaction between the four-way junction and the lower part of P7.

The Major group fed the stems P2 and P4 as constraints to MC-Fold (Parisien and Major 2008). Various alternative secondary structural elements were indicated by Barrick and Breaker, especially between P7 and P11. The Major group identified a sequence with a potential to adopt a kink-turn, similar to the kink-turn predicted in the snoRNA U3 C′/D box (Rozhdestvensky et al. 2003). This kink-turn has a GA tandem and an asymmetric loop. We decided to assume its formation by adding it to the constraints using a mask in MC-Fold:

GGGAGU-GCGAGGAUC
((((((-))...))))

The 3D model was built by using the T-loop crystal structure of a tRNA (PDB 1EVV). The kink-turn area was modeled after Kt-7 (PDB ID 3CC2 of the 23S rRNA of *Haloarcula Marismortui*). The remaining parts and the final assembly were modeled using MC-Sym (major.iric.ca/Web/mctools). The models generated by MC-Sym were minimized up to the “brushup” level of the MC-Pipeline. The selection of the candidate models was based on “Score” values, a homemade all-atoms force-field which is part of the “Analysis” module of the MC-pipeline.

### Xiao group

The Xiao group used 3dRNA web server (http://122.205.6.127/3dRNA/3dRNA.html) (Zhao et al. 2012) to complete the prediction for Problems 5 and 6. 3dRNA builds tertiary structure of an RNA molecule by assembling three-dimensional (3D) structural templates of its secondary structure elements, including helix, hairpin loop, internal loop, bulge loop, and multiway junction. The 3D templates are from a library extracted from experimental RNA structures. In addition, 3dRNA can build different models for an RNA molecule by using different templates for each of secondary structure elements.

In the prediction for Problems 5 and 6, they first predicted the secondary structures of the submitted sequences by using Mfold (Zuker 2003) and RNAfold (Hofacker et al. 1994) and picked out the optimal prediction. Then, the sequences and predicted secondary structures were submitted to 3dRNA web server and a set of structural models was generated. Finally, these structural models were ranked with a scoring function 3dRNAscore (data not shown) and the lowest energy structures were selected as the candidate structures.

For Problem 5, the Xiao group used Mfold server (Zuker 2003) and RNAfold (Hofacker et al. 1994) to predict the secondary structure. The Mfold web server gave 10 secondary structures consisting of an optimal and nine suboptimal folds. The RNAfold web server gave an optimal secondary structure by minimum free energy and an optimal secondary structure by thermodynamic ensemble. They used both the Mfold and RNAfold optimal folds to predict the tertiary structure. One of the optimal secondary structure is a three-way-junction structure whose secondary structure is (..().().....()). This three-way junction had no 3D templates in the library but 3dRNA could automatically search the library and pick out a nearest three-way junction, whose secondary structure is (..()..()....()). In this case, the loop was extracted from a ribozyme fragment 1GID. 3dRNA then made deletion and insertion operations on it to match the secondary structure needed, i.e., (..().().....()). After that, the 3D templates of other secondary structural elements were automatically searched for by the searching module of 3dRNA and then assembled to a whole all-atom structure by the assembling module of 3dRNA. Finally, the structure was refined by AMBER energy minimization. All calculations were performed on an Intel S5500BC server (Intel(R) Xeon(R) CPU E5620 @ 2.40 GHz). The template searching and assembling process took ∼2 min. The AMBER energy minimization took ∼3 min.

As above, for Problem 6, the Xiao group used Mfold and RNAfold to predict the secondary structure. The optimal secondary structure predicted by Mfold is a four-way junction structure and the optimal secondary structure predicted by RNAfold is made of three-way junction structures. The barrier for 3dRNA is the lack of the 3D templates for the four-way junction: (().....()....().......). As previously, 3dRNA searched the templates library for the nearest loop. A four-way junction with the secondary structure (..()........()......()..) was picked out. It was extracted from rRNA 1C2W. After deletion and insertion operations, a 3D template of four-way junction with the secondary structure (().....()....().......) was created. After that, the whole tertiary structure was assembled smoothly. Ten models were predicted for each of the optimal predicted secondary structures and then scored by 3dRNAscore. The lowest energy model was selected as the candidate and further refined with AMBER energy minimization. All calculations were performed on an Intel S5500BC server (Intel(R) Xeon(R) CPU E5620 @ 2.40 GHz). The whole template searching and assembling process took <3 sec this time as the 3dRNA has been reimplemented. The AMBER energy minimization process took 38 sec.

## DISCUSSION

Except in some cases (Levitt 1969; Michel and Westhof 1990), RNA 3D structure prediction has historically lagged behind protein structure prediction, although RNA Watson–Crick pairing (secondary structure) is simpler to predict than, for example, β−sheet pairings in proteins. Nevertheless, compared to protein structure, RNA has more degrees of freedom. In addition, despite the limited number of non-Watson–Crick base pairs that simplifies analysis and inspection of tertiary structure, these non-Watson–Crick base pairs are difficult to recognize but central to the three-dimensional architecture of folded RNA molecules. The backbone of a nucleotide has six rotatable bonds, while an amino acid includes three (and the ω dihedral angle is generally fixed ∼180° in peptide plane). Therefore, the RNA conformational landscape is potentially much larger and the three-dimensional structure prediction of RNAs with 100 nt is comparable, in terms of the number of degrees of freedom and molecular weight, to the challenge of modeling proteins of 200–300 aa. Although the structures in this round of RNA-Puzzles are large, topologies of the best predictions are not extreme compared with the native structures. This is a positive signal for RNA structure modeling.

In the current stage, most predictions can achieve good accuracy on Watson–Crick base pairs, while non-Watson–Crick interactions remain an open challenge that constitute an important bottleneck in RNA structure modeling. To improve non-WC pair prediction, RNA module prediction should be emphasized, since RNA modules are stable in structure but difficult to predict. Programs such as RMDetect (Cruz and Westhof 2011) could help in predicting RNA modules and improve the non-WC interaction prediction, e.g., for the K-turn in the adenosylcobalamin riboswitch which was recognized by modelers but not automatically. Unlike the numerous structures available for proteins, the number of RNA structures solved by crystallization is still limited and the available conformational space of RNA folding is far from complete. The prediction of non-WC pair and stacking could also be improved with the increase of known RNA structures and a complete search for RNA modules.

Other than module prediction programs, easy and fast experiments can provide direct constraints in structure modeling. In the protein structure prediction trials CASP Round X (Moult et al. 2014), a new category of “contact-assisted” prediction was proposed. Experimental data such as NMR, chemical shift, cross-linking, and surface labeling have been proved to be instrumental. Previously, contacts inferred from evolutionary information also achieved success in protein structure modeling (Morcos et al. 2011) but, at the time of writing, they still have not had an impact in blind structure prediction tests (Moult et al. 2014). Nevertheless, these explorations have revealed a trend in structure modeling: With the help of simple experimental constraints, structure modeling could achieve the application level in providing structural information for biological problems, even if no homologous structures are available. According to the three large RNA structures in this round of RNA-Puzzles, the modeling of RNA topology structures are already close to native, and the relative orientation between T-box and tRNA structures are recovered at a resolution (6.8 Å) comparable to the spacing between nucleotides.

Although the best predictions are similar to the topology of native RNA structures, some beauties in native structure topologies cannot be captured. As an example, the dramatic hole in the ring structure formed by two helices in Problem 5 was not described at even nucleotide resolution by any prediction model. Current fast experiments can only help in detecting local and detailed interactions rather than global architectures determined by long-range contacts. These methods are mainly sensitive to detailed contacts but are largely uninformative as to global information such as holes. For consistent refinement to higher resolution 3D models that predict these striking features, we still need deeper understanding of RNA structure and/or new fast experimental tools.

Currently, the major challenges in RNA structure prediction lie in (1) further improvement of algorithms that incorporate simple experimental data (contact-assisted data), (2) structure optimization to alleviate atomic clashes and improve accuracy, and (3) accumulation of comprehensive RNA structure knowledge with the help of database increases and automated structural bioinformatic tools. The surprising high values for the clash scores in several otherwise respectable models led to attempts to improve the clash score values by rerefinement. The Das group had run ERRASER (Chou et al. 2013), but ERRASER only works to find solutions with each nucleotide within ∼2 Å RMSD of the starting solution. Many of the derived models used fragments of other crystal structures that did not fit well together. The relief of chainbreaks and clashes require bigger changes than ERRASER can currently handle. After an exchange of the previous versions of this article, the Bujnicki group ran their refinement method QRNAS on the Das models (all 17 models submitted for the three Problems). QRNAS is essentially a reimplementation of AMBER with additional regularization and it is used as the final element in the Bujnicki modeling pipeline. In all cases, a dramatic reduction of Clash Scores was obtained; in 10 models even down to zero. Only in three cases the Clash Scores remained larger than 4; however, these models had initial Clash Scores of nearly 30. Supplemental Table S2 shows the values of all the metrics used for comparisons and most of them display an improvement or at least no worsening. However, the bond angle deviations increase severely in all cases, a not so surprising result since that parameter was kept free during optimization. Thus, further work is required for resolving clashes in automatically derived models.

As attested by the number of coauthors involved in these three RNA Puzzles in most modeling groups, the automaticity of the 3D structure prediction process still requires a major investment in computer science and in the development of user-friendly and straightforward computer tools. Therefore, in order to make RNA 3D structure prediction available to the biological community in solving biological problems, we encourage web servers for automatic RNA 3D structure prediction. Such web servers should take query sequences, probably together with simple experimental data, and return possible RNA 3D coordinates. As described, several groups have already advanced in this direction. As inspiration, in recent years, servers have largely caught up with human expert groups in protein structure prediction (Moult et al. 2014), and it will be interesting to see if the RNA community can accomplish the same.

Finally, in the present comparisons, it is assumed that the crystal structure is the relevant and correct target. Crystallographic structures constitute highly relevant models representing with high precision and accuracy particular experiments and conditions. However, not all segments of crystallized structures are at the same level of accuracy, because of resolution issues, disorder, or high segmental mobilities (as represented by the thermal B-factors). For those segments, the uncertainty of the reference structure is a real question. A meaningful comparison would thus require that the prediction programs derive also a theoretical B-factor for the nucleotides representing some aspects of the uncertainty in the prediction. Preliminary results indicate that regions with high experimental B-factors correlate with regions in disagreement with the rest of the structure (regions in red color in the deformation profiles). Thoughtful weighted comparisons need to be developed to address these issues of molecular dynamics during comparisons between crystal structures and predicted models.

## SUPPLEMENTAL MATERIAL

Supplemental material is available for this article.

## Supplementary Material

Supplemental Material
