# Supplementary material for: RNA-Puzzles Round II: assessment of RNA structure prediction programs applied to three large RNA structures
Source: RNA. 2015 Jun;21(6):1066–84. doi: 10.1261/rna.049502.114 (PMC4436661; doi:10.1261/rna.049502.114)
Supplement: Supplemental Material [file supp_21_6_1066__index.html]

RNA-Puzzles Round II: assessment of RNA structure prediction programs applied to three large RNA structures — RNA-Puzzles Round II: assessment of RNA structure prediction programs applied to three large RNA structures — Supplemental Material 

# *RNA-Puzzles* Round II: assessment of RNA structure prediction programs applied to three large RNA structures

## Supplemental Material

**Files in this Data Supplement:**

- Supp Figure S4.tif
- Supp Tables & Legends.docx
- Supp Figure S3.tif
- Supp Figure S5.tif
- Supp Figure S6.tif
- Supp Figure S1.tif
- Supp Figure S2.tif
