# Supplementary figures and images for: RNA-Puzzles Round II: assessment of RNA structure prediction programs applied to three large RNA structures
Source: RNA. 2015 Jun;21(6):1066–84. doi: 10.1261/rna.049502.114 (PMC4436661; doi:10.1261/rna.049502.114)

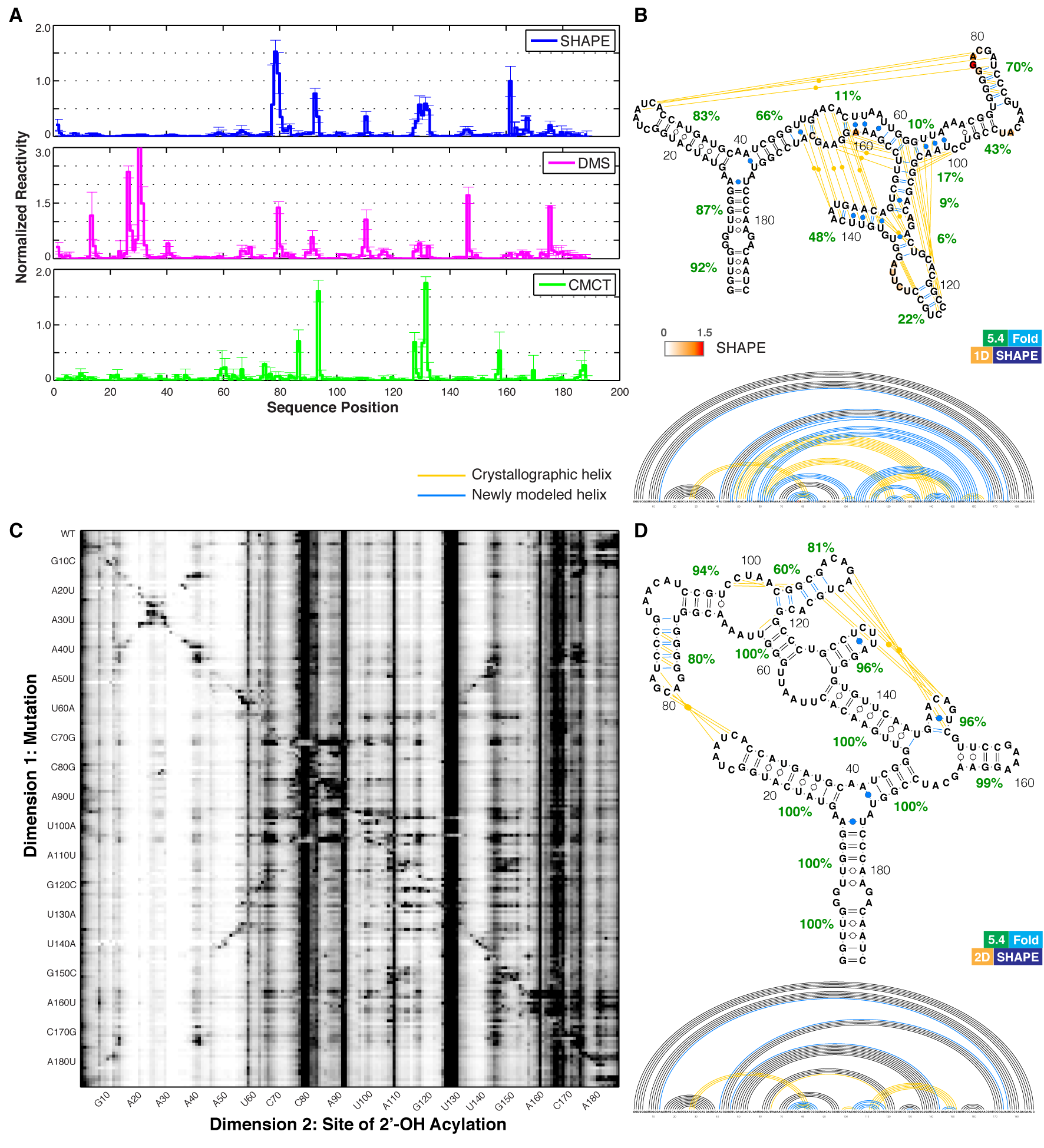

Supplement: Supplemental Material [file supp_049502.114_fig-S4.tif]

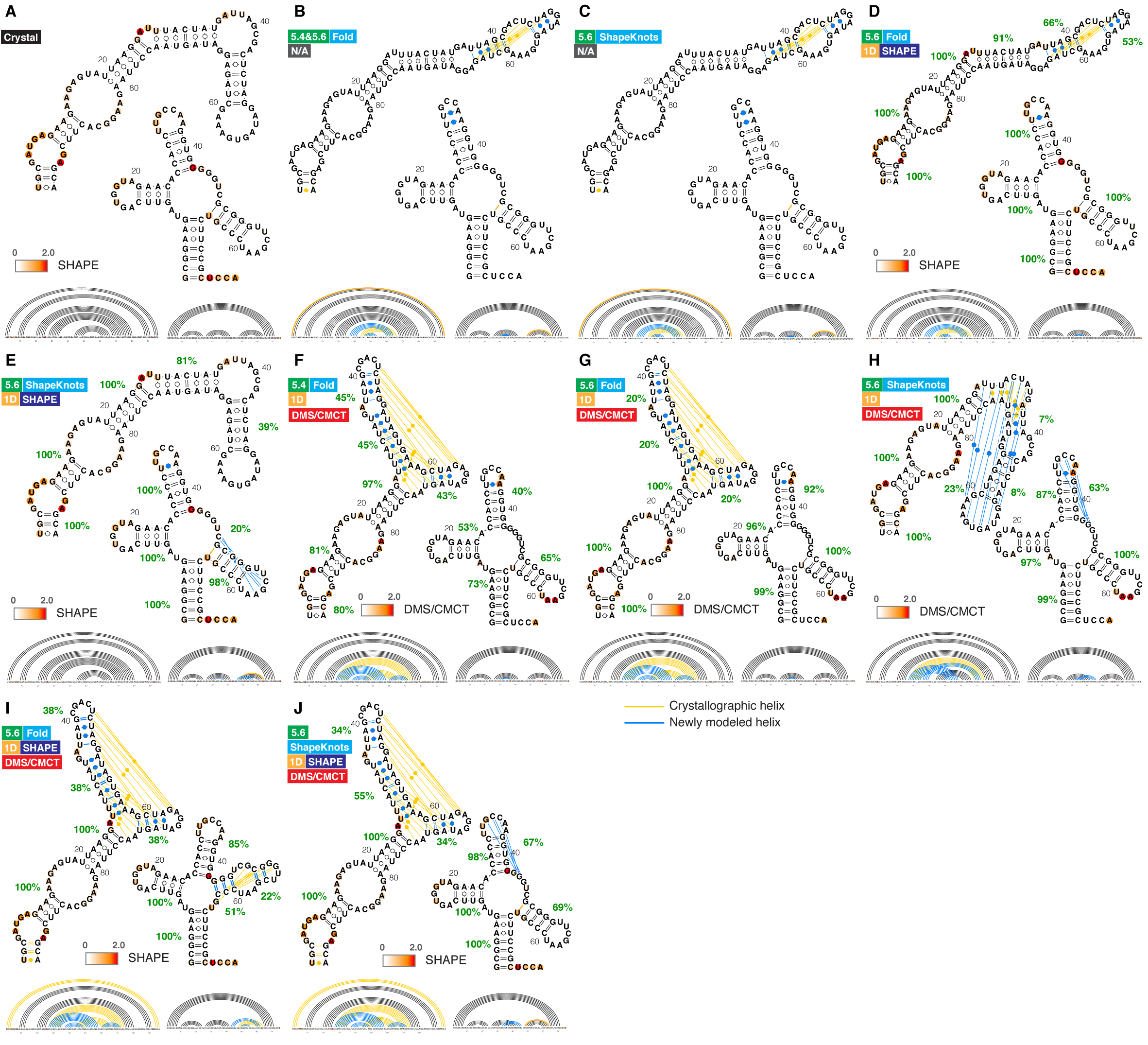

Supplement: Supplemental Material [file supp_049502.114_fig-S3.tif]

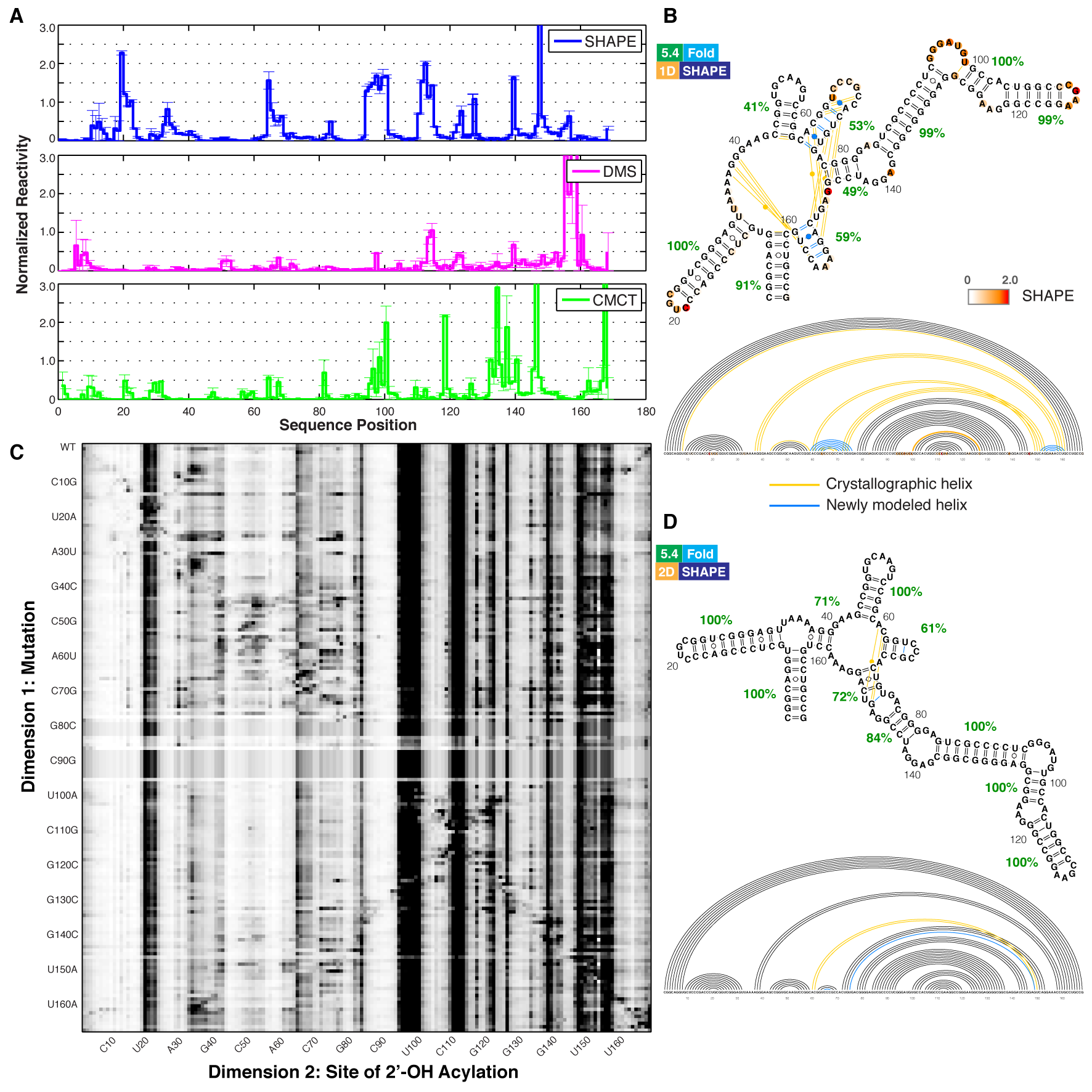

Supplement: Supplemental Material [file supp_049502.114_fig-S5.tif]

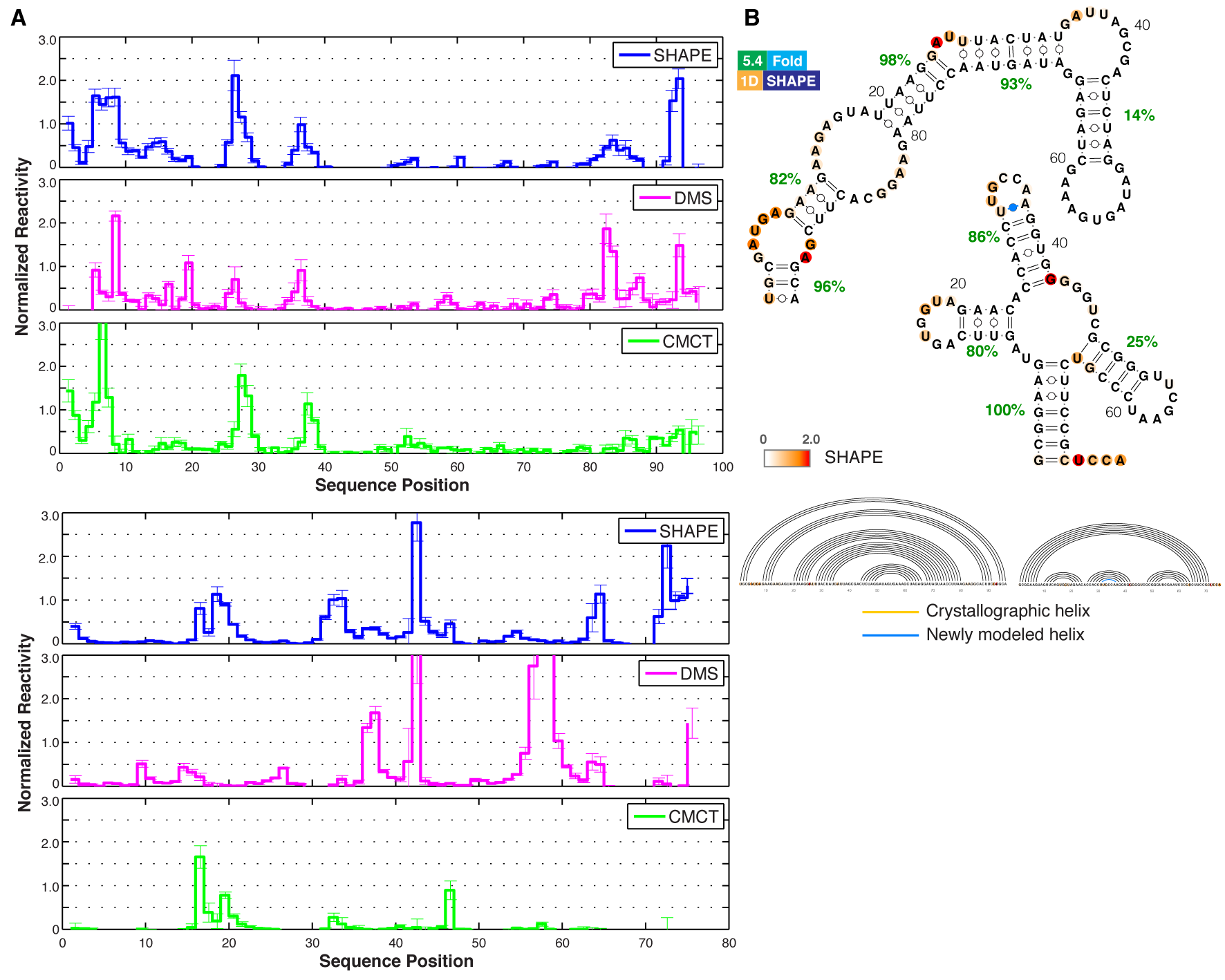

Supplement: Supplemental Material [file supp_049502.114_fig-S6.tif]

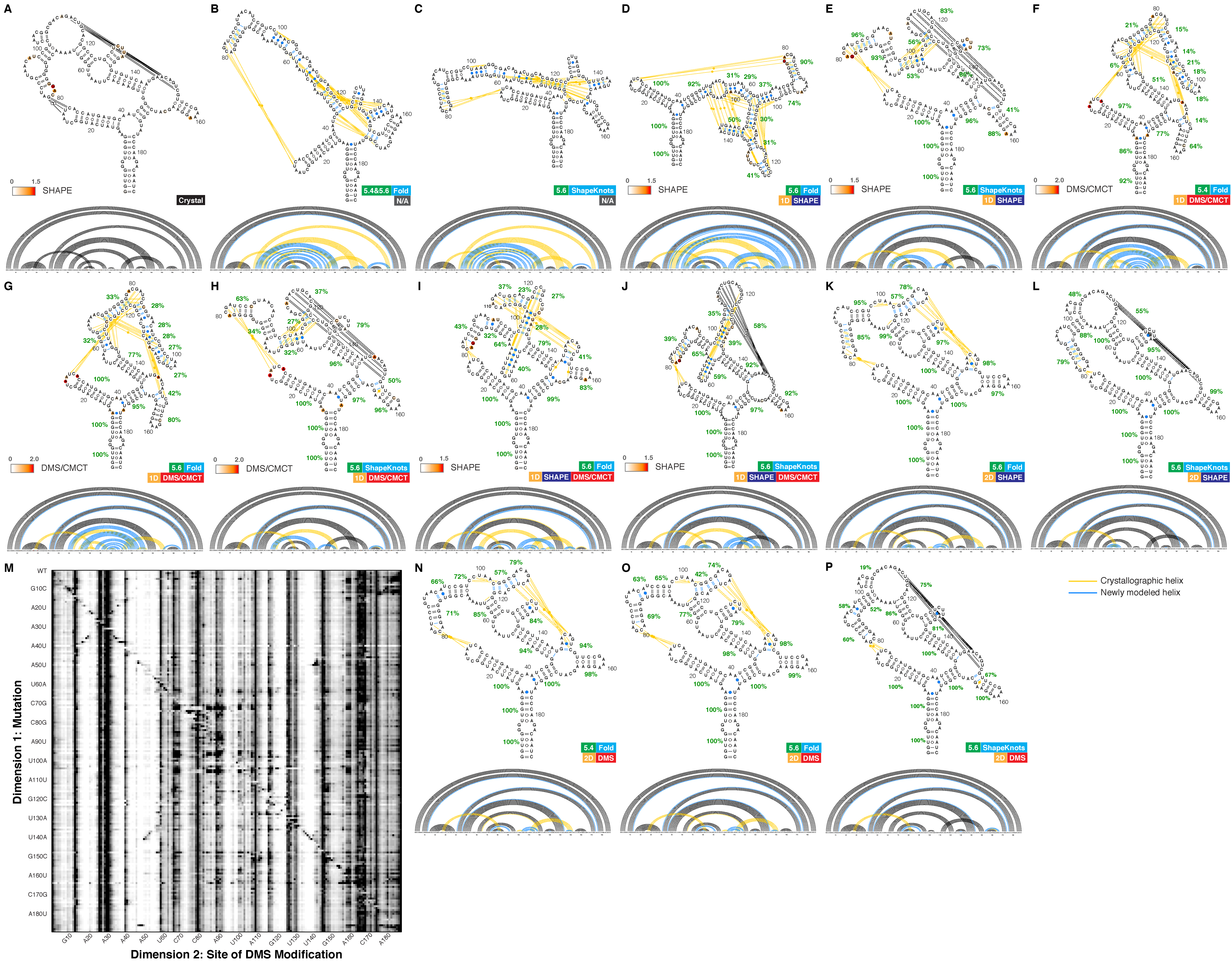

Supplement: Supplemental Material [file supp_049502.114_fig-S1.tif]

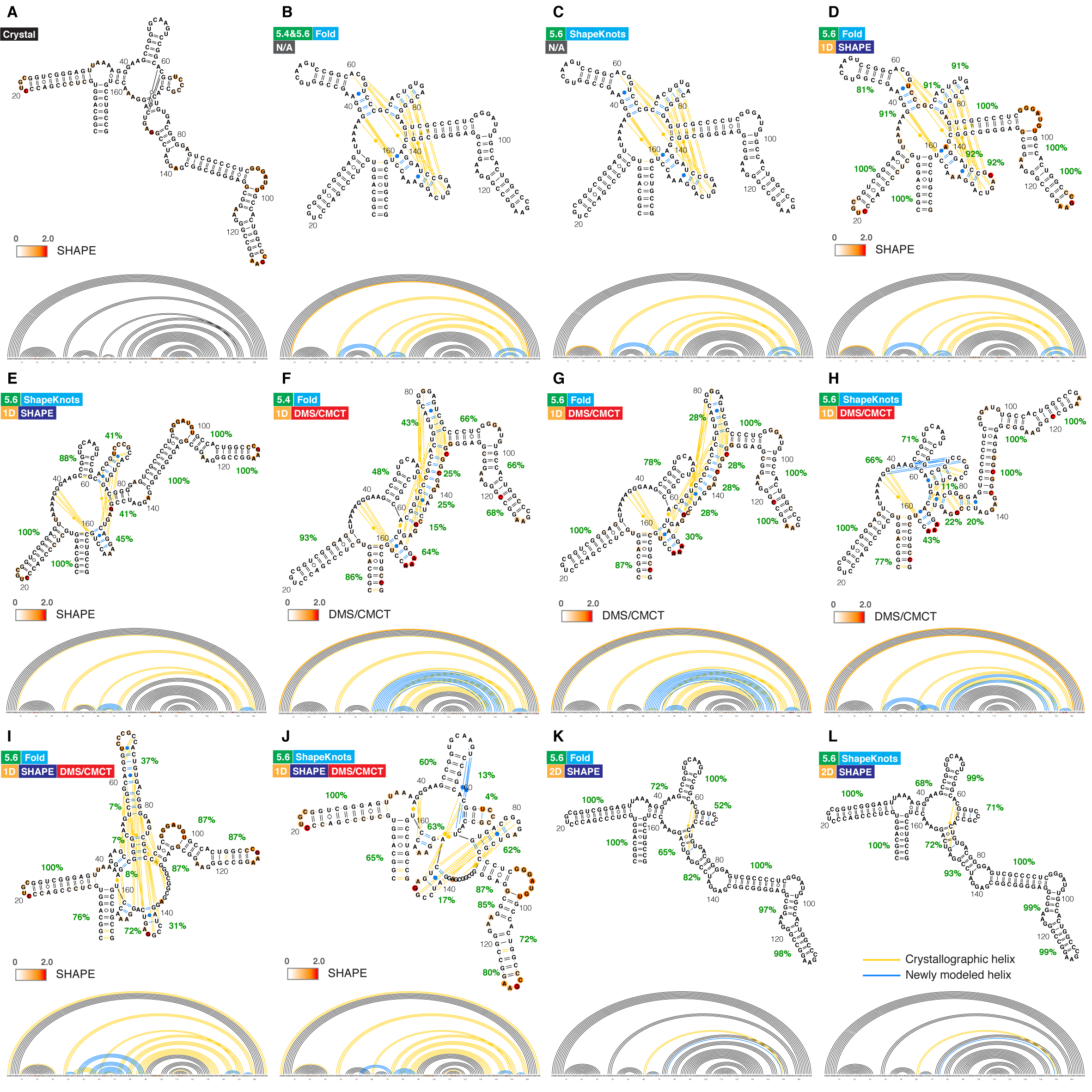

Supplement: Supplemental Material [file supp_049502.114_fig-S2.tif]
