# Supplementary material for: RNA-Puzzles Round II: assessment of RNA structure prediction programs applied to three large RNA structures
Source: RNA. 2015 Jun;21(6):1066–84. doi: 10.1261/rna.049502.114 (PMC4436661; doi:10.1261/rna.049502.114)
Supplement: Supplemental Material [file supp_049502.114_SuppTables_Legends.docx]

# *RNA-Puzzles* Round II: Assessment of RNA structure prediction programs applied to three large RNA structures

Zhichao Miao^1^, Ryszard W. Adamiak^2^, Marc-Frédérick Blanchet^3^, Michal Boniecki^4^, Janusz M. Bujnicki^4,5^, Shi-Jie Chen^6^, Clarence Cheng^7^, Grzegorz Chojnowski^4^, Fang-Chieh Chou^7^, Pablo Cordero^7^, José Almeida Cruz^1^, Adrian Ferre-D'Amare^8^, Rhiju Das^7^, Feng Ding^9^, Nikolay V. Dokholyan^10^, Stanislaw Dunin-Horkawicz^4^, Wipapat Kladwang^7^, Andrey Krokhotin^10^, Grzegorz Lach^4^, Marcin Magnus^4^, François Major^3^, Thomas H. Mann^7^, Benoît Masquida^11^, Dorota Matelska^4^, Mélanie Meyer^12^, Alla Peselis^13^, Mariusz Popenda^2^, Katarzyna J. Purzycka^2^, Alexander Serganov^13^, Juliusz Stasiewicz^4^, Marta Szachniuk^15^, Arpit Tandon^10^, Siqi Tian^7^, Jian Wang^14^, Yi Xiao^14^, Xiaojun Xu^6^, Jinwei Zhang^8^, Peinan Zhao^6^, Tomasz Zok^15^ and Eric Westhof^1,*^

^1^Architecture et Réactivité de l'ARN, Université de Strasbourg, Institut de biologie moléculaire et cellulaire du CNRS, 67000 Strasbourg France; ^2^Department of Structural Chemistry and Biology of Nucleic Acids, Structural Chemistry of Nucleic Acids Laboratory, Institute of Bioorganic Chemistry, Polish Academy of Sciences, Poznan, Poland; ^3^Institute for Research in Immunology and Cancer (IRIC), Department of Computer Science and Operations Research, Université de Montréal, Montréal, Québec H3C 3J7, Canada; ^4^Laboratory of Bioinformatics and Protein Engineering, International Institute of Molecular and Cell Biology in Warsaw, 02-109 Warsaw, Poland; ^5^Laboratory of Bioinformatics, Institute of Molecular Biology and Biotechnology, Faculty of Biology, Adam Mickiewicz University, 61-614 Poznan, Poland^; 6^Department of Physics and Astronomy, Department of Biochemistry, University of Missouri Informatics Institute, University of Missouri-Columbia, MO 65211, U.S.A.; ^7^Department of Physics, Stanford University, Stanford, California 94305, USA; ^8^National Heart, Lung and Blood Institute, 50 South Drive, MSC 8012, Bethesda, Maryland 20892-8012, USA; ^9^Department of Physics and Astronomy at Clemson University, College of Engineering and Science, USA; ^10^Department of Biochemistry and Biophysics, University of North Carolina, School of Medicine, Chapel Hill, North Carolina, USA;^11^Génétique Moléculaire Génomique Microbiologie, Institut de physiologie et de la chimie biologique, 21 rue René Descartes 67084 Strasbourg, France;^12^Institut de génétique et de biologie moléculaire et cellulaire, 1 Rue Laurent Fries, 67400 Strasbourg, France; ^13^Department of Biochemistry and Molecular Pharmacology, New York University School of Medicine, New York, New York, USA, ^14^Department of Physics, Huazhong University of Science and Technology, Wuhan, China; ^15^Poznan University of Technology, Institute of Computing Science, Poznan, Poland.

**Supplementary information**

| **TABLE S1**.Summary of experimental data | |  | base-pair | | helix | |
| --- | --- | --- | --- | --- | --- | --- |
|  |  |  | sensitivity | ppv | sensitivity | ppv |
| Puzzle 5 | No data | 5.4 Fold | 50.0% | 48.4% | 46.2% | 42.9% |
|  |  | 5.6 Fold | 50.0% | 48.4% | 46.2% | 42.9% |
|  |  | 5.6 ShapeKnots | 50.0% | 50.0% | 46.2% | 42.9% |
|  | 1D SHAPE | 5.4 Fold | 45.0% | 41.5% | 38.5% | 35.7% |
|  |  | 5.6 Fold | 45.0% | 41.5% | 69.2% | 35.7% |
|  |  | 5.6 ShapeKnots | 78.3% | 72.3% | 46.2% | 69.2% |
|  | 1D DMS/CMCT | 5.4 Fold | 58.3% | 53.9% | 46.2% | 42.9% |
|  |  | 5.6 Fold | 58.3% | 53.9% | 76.9% | 42.9% |
|  |  | 5.6 ShapeKnots | 81.7% | 79.0% | 61.5% | 76.9% |
|  | 1D SHAPE/DMS/CMCT | 5.6 Fold | 66.7% | 60.6% | 61.5% | 53.3% |
|  |  | 5.6 ShapeKnots | 73.3% | 67.7% | 69.2% | 66.7% |
|  | 2D SHAPE | 5.4 Fold | 76.7% | 74.2% | 69.2% | 69.2% |
|  |  | 5.6 Fold | 76.7% | 74.2% | 84.6% | 69.2% |
|  |  | 5.6 ShapeKnots | 90.0% | 85.7% | 92.3% | 91.7% |
|  | 2D DMS | 5.4 Fold | 81.7% | 77.8% | 76.9% | 71.4% |
|  |  | 5.6 Fold | 81.7% | 77.8% | 76.9% | 71.4% |
|  |  | 5.6 ShapeKnots | 93.3% | 86.2% | 92.3% | 85.7% |
| Puzzle 6 | No data | 5.4 Fold | 73.7% | 73.7% | 54.6% | 54.6% |
|  |  | 5.6 Fold | 73.7% | 73.7% | 54.6% | 54.6% |
|  |  | 5.6 ShapeKnots | 73.7% | 73.7% | 54.6% | 54.6% |
|  | 1D SHAPE | 5.4 Fold | 77.2% | 81.5% | 63.6% | 77.8% |
|  |  | 5.6 Fold | 73.7% | 73.7% | 54.6% | 54.6% |
|  |  | 5.6 ShapeKnots | 79.0% | 81.8% | 63.6% | 77.8% |
|  | 1D DMS/CMCT | 5.4 Fold | 63.2% | 64.3% | 54.6% | 50.0% |
|  |  | 5.6 Fold | 63.2% | 64.3% | 54.6% | 50.0% |
|  |  | 5.6 ShapeKnots | 71.9% | 70.7% | 54.6% | 54.6% |
|  | 1D SHAPE/DMS/CMCT | 5.6 Fold | 49.1% | 57.1% | 36.4% | 33.3% |
|  |  | 5.6 ShapeKnots | 63.2% | 72.0% | 63.6% | 58.3% |
|  | 2D SHAPE | 5.4 Fold | 96.5% | 96.5% | 90.9% | 90.9% |
|  |  | 5.6 Fold | 96.5% | 96.5% | 90.9% | 90.9% |
|  |  | 5.6 ShapeKnots | 96.5% | 96.5% | 90.9% | 90.9% |
| Puzzle 10 | No data | 5.4 Fold | 83.0% | 79.6% | 88.9% | 80.0% |
|  |  | 5.6 Fold | 83.0% | 79.6% | 88.9% | 80.0% |
|  |  | 5.6 ShapeKnots | 83.0% | 79.6% | 88.9% | 80.0% |
|  | 1D SHAPE | 5.4 Fold | 100.0% | 97.9% | 100.0% | 100.0% |
|  |  | 5.6 Fold | 87.2% | 82.0% | 88.9% | 80.0% |
|  |  | 5.6 ShapeKnots | 97.9% | 92.0% | 100.0% | 90.0% |
|  | 1D DMS/CMCT | 5.4 Fold | 72.3% | 66.7% | 77.8% | 70.0% |
|  |  | 5.6 Fold | 72.3% | 66.7% | 77.8% | 70.0% |
|  |  | 5.6 ShapeKnots | 85.1% | 71.4% | 88.9% | 72.7% |
|  | 1D SHAPE/DMS/CMCT | 5.6 Fold | 55.3% | 55.3% | 55.6% | 50.0% |
|  |  | 5.6 ShapeKnots | 63.8% | 61.2% | 66.7% | 60.0% |

| **Table S2.** Structure Refinement based on Das lab models done by Bujnicki lab. | | | | | | | | | | | | | | |
| --- | --- | --- | --- | --- | --- | --- | --- | --- | --- | --- | --- | --- | --- | --- |
| Problem | Lab | Num | RMSD | P-value | DI | INF | INF_wc | INF_nwc | INF_stacking | clash | pct_badbonds | pct_resbadbonds | pct_badangles | pct_resbadangles |
| 5 | DasRef^*^ | 1 | 9.96 | 0.00E+000 | 13.002 | 0.766 | 0.919 | 0.256 | 0.766 | 0 | 0.7 | 7.45 | 8.33 | 100 |
| 5 | DasRef | 2 | 9.165 | 0.00E+000 | 11.94 | 0.768 | 0.908 | 0.344 | 0.757 | 0.17 | 0.25 | 1.6 | 7.07 | 100 |
| mean |  |  |  |  |  |  |  |  |  |  | 0.475 | 4.525 | 7.7 | 100 |
| 5 | Das | 1 | 9.948 | 0.00E+000 | 13.148 | 0.757 | 0.919 | 0.256 | 0.751 | 9.44 | 0.74 | 9.57 | 1.48 | 27.66 |
| 5 | Das | 2 | 9.152 | 0.00E+000 | 12.019 | 0.761 | 0.906 | 0.334 | 0.751 | 6.79 | 0.49 | 6.38 | 1.52 | 30.85 |
| mean |  |  |  |  |  |  |  |  |  |  | 0.615 | 7.975 | 1.5 | 29.255 |
|  |  |  |  |  |  |  |  |  |  |  |  |  |  |  |
| Problem | Lab | Num | RMSD | P-value | DI | INF | INF_wc | INF_nwc | INF_stacking | clash | pct_badbonds | pct_resbadbonds | pct_badangles | pct_resbadangles |
| 6 | DasRef | 1 | 14.504 | 3.23E-009 | 19.022 | 0.762 | 0.897 | 0.433 | 0.746 | 3.85 | 0.41 | 2.38 | 7.88 | 100 |
| 6 | DasRef | 2 | 13.683 | 1.92E-010 | 17.889 | 0.765 | 0.905 | 0.361 | 0.755 | 0 | 0 | 0 | 7.04 | 100 |
| 6 | DasRef | 3 | 15.778 | 1.73E-007 | 21.019 | 0.751 | 0.889 | 0.334 | 0.744 | 0.18 | 0.05 | 0.6 | 7.5 | 100 |
| 6 | DasRef | 4 | 11.714 | 9.59E-014 | 15.595 | 0.751 | 0.897 | 0.416 | 0.731 | 5.32 | 0.78 | 5.95 | 9.34 | 100 |
| 6 | DasRef | 5 | 15.862 | 2.22E-007 | 20.509 | 0.773 | 0.897 | 0.433 | 0.763 | 0 | 0 | 0 | 6.8 | 100 |
| 6 | DasRef | 6 | 12.421 | 1.68E-012 | 16.464 | 0.754 | 0.897 | 0.433 | 0.734 | 3.85 | 0.37 | 1.79 | 7.77 | 100 |
| 6 | DasRef | 7 | 17.959 | 5.12E-005 | 24.221 | 0.741 | 0.877 | 0.316 | 0.732 | 0 | 0 | 0 | 7.15 | 100 |
| 6 | DasRef | 8 | 17.97 | 5.26E-005 | 23.451 | 0.766 | 0.897 | 0.416 | 0.755 | 0 | 0.82 | 5.95 | 8.32 | 100 |
| 6 | DasRef | 9 | 15.107 | 2.26E-008 | 19.785 | 0.764 | 0.905 | 0.316 | 0.755 | 0 | 0.05 | 0.6 | 6.72 | 100 |
| 6 | DasRef | 10 | 29.226 | 9.91E-001 | 39.946 | 0.732 | 0.897 | 0.312 | 0.718 | 0.37 | 0.41 | 2.38 | 8.02 | 100 |
| mean |  |  |  |  |  |  |  |  |  |  | 0.289 | 1.965 | 7.654 | 100 |
| 6 | Das | 1 | 14.478 | 2.96E-009 | 19.893 | 0.728 | 0.885 | 0.333 | 0.705 | 23.85 | 0.64 | 8.33 | 0.87 | 10.71 |
| 6 | Das | 2 | 13.627 | 1.57E-010 | 18.774 | 0.726 | 0.885 | 0.347 | 0.705 | 17.24 | 0.6 | 7.14 | 0.84 | 10.12 |
| 6 | Das | 3 | 15.752 | 1.60E-007 | 21.759 | 0.724 | 0.874 | 0.217 | 0.72 | 17.05 | 0.55 | 7.14 | 0.62 | 9.52 |
| 6 | Das | 4 | 11.699 | 9.02E-014 | 16.151 | 0.724 | 0.885 | 0.316 | 0.702 | 23.48 | 0.64 | 8.33 | 0.92 | 11.31 |
| 6 | Das | 5 | 15.834 | 2.04E-007 | 21.147 | 0.749 | 0.885 | 0.361 | 0.738 | 13.39 | 0.37 | 4.76 | 0.65 | 8.93 |
| 6 | Das | 6 | 12.405 | 1.58E-012 | 17.08 | 0.726 | 0.885 | 0.333 | 0.702 | 24.59 | 0.64 | 8.33 | 0.92 | 11.9 |
| 6 | Das | 7 | 17.875 | 4.22E-005 | 24.363 | 0.734 | 0.869 | 0.237 | 0.731 | 13.21 | 0.55 | 7.14 | 0.6 | 8.93 |
| 6 | Das | 8 | 17.956 | 5.08E-005 | 24.06 | 0.746 | 0.877 | 0.334 | 0.743 | 13.02 | 0.41 | 5.36 | 0.62 | 8.33 |
| 6 | Das | 9 | 15.048 | 1.88E-008 | 20.942 | 0.719 | 0.885 | 0.347 | 0.694 | 17.98 | 0.6 | 7.14 | 0.79 | 10.12 |
| 6 | Das | 10 | 29.182 | 9.91E-001 | 41.226 | 0.708 | 0.877 | 0.25 | 0.698 | 19.82 | 0.64 | 7.74 | 0.62 | 6.55 |
| mean |  |  |  |  |  |  |  |  |  |  | 0.564 | 7.141 | 0.745 | 9.642 |
|  |  |  |  |  |  |  |  |  |  |  |  |  |  |  |
| Problem | Lab | Num | RMSD | P-value | DI | INF | INF_wc | INF_nwc | INF_stacking | clash | pct_badbonds | pct_resbadbonds | pct_badangles | pct_resbadangles |
| 10 | DasRef | 1 | 7.64 | 0.00E+000 | 8.876 | 0.861 | 0.929 | 0.7 | 0.861 | 0 | 0 | 0 | 6.67 | 98.83 |
| 10 | DasRef | 2 | 10.539 | 1.50E-015 | 12.191 | 0.864 | 0.938 | 0.802 | 0.847 | 0 | 0 | 0 | 6.59 | 98.83 |
| 10 | DasRef | 3 | 6.837 | 0.00E+000 | 8.157 | 0.838 | 0.936 | 0.717 | 0.823 | 0 | 0 | 0 | 6.46 | 98.83 |
| 10 | DasRef | 4 | 7.077 | 0.00E+000 | 8.305 | 0.852 | 0.938 | 0.717 | 0.842 | 0 | 0 | 0 | 6.7 | 98.83 |
| 10 | DasRef | 5 | 10.482 | 1.17E-015 | 11.944 | 0.878 | 0.938 | 0.778 | 0.87 | 0 | 0 | 0 | 6.73 | 98.83 |
| mean |  |  |  |  |  |  |  |  |  |  | 0 | 0 | 6.63 | 98.83 |
| 10 | Das | 1 | 7.58 | 0.00E+000 | 9.199 | 0.824 | 0.92 | 0.7 | 0.811 | 11.64 | 0.36 | 4.09 | 0.56 | 8.77 |
| 10 | Das | 2 | 10.447 | 9.99E-016 | 12.588 | 0.83 | 0.929 | 0.778 | 0.803 | 11.64 | 0.36 | 4.09 | 0.56 | 8.19 |
| 10 | Das | 3 | 6.803 | 0.00E+000 | 8.365 | 0.813 | 0.946 | 0.7 | 0.786 | 11.09 | 0.41 | 4.68 | 0.64 | 9.36 |
| 10 | Das | 4 | 7.062 | 0.00E+000 | 8.539 | 0.827 | 0.948 | 0.684 | 0.809 | 10.73 | 0.41 | 4.68 | 0.56 | 9.94 |
| 10 | Das | 5 | 10.417 | 8.88E-016 | 12.295 | 0.847 | 0.948 | 0.778 | 0.823 | 10.91 | 0.27 | 2.92 | 0.45 | 7.02 |
| mean |  |  |  |  |  |  |  |  |  |  | 0.362 | 4.092 | 0.554 | 8.656 |
|  |  |  |  |  |  |  |  |  |  |  |  |  |  |  |
| pct_badbonds: percentage of bad bonds | | | | |  |  |  |  |  |  |  |  |  |  |
| pct_resbadbonds: percentage of residues with bad bonds | | | | | |  |  |  |  |  |  |  |  |  |
| pct_badangles: percentage of bad angles | | | | |  |  |  |  |  |  |  |  |  |  |
| pct_resbadangles: percentage of residues with bad angles | | | | | |  |  |  |  |  |  |  |  |  |

*The refinements were done by Bujnicki lab to alleviate the atomic clashes in models of Das lab and named as DasRef.

**Figure S1. Chemical mapping data and secondary structure predictions of RNA Puzzle 5.**

1. Secondary structure from crystallographic structures.
2. Secondary structure prediction using no experimental data with RNAstructure 5.4 or 5.6 *Fold*. Nucleotides are colored according with SHAPE reactivities. Crystallographic pairings missing in this model and new non-crystallographic pairings are drawn as yellow and blue lines, respectively. Percentage labels give bootstrap support values.
3. Secondary structure prediction using no data with RNAstructure 5.6 *ShapeKnots*.
4. Secondary structure prediction using 1D SHAPE data with RNAstructure 5.6 *Fold*.
5. Secondary structure prediction using 1D SHAPE data with RNAstructure 5.6 *ShapeKnots*.
6. Secondary structure prediction using 1D DMS/CMCT data with RNAstructure 5.4 *Fold*.
7. Secondary structure prediction using 1D DMS/CMCT data with RNAstructure 5.6 *Fold*.
8. Secondary structure prediction using 1D DMS/CMCT data with RNAstructure 5.6 *ShapeKnots*.
9. Secondary structure prediction using 1D SHAPE and DMS/CMCT data with RNAstructure 5.6 *Fold*.
10. Secondary structure prediction using 1D SHAPE and DMS/CMCT data with RNAstructure 5.6 *ShapeKnots*.
11. Secondary structure prediction using 2D SHAPE M^2^ data with RNAstructure 5.6 *Fold*.
12. Secondary structure prediction using 2D SHAPE M^2^ data with RNAstructure 5.6 *ShapeKnots*.
13. Mutate-and-map (M^2^) dataset probed by the DMS.
14. Secondary structure prediction using 2D SHAPE M^2^ data with RNAstructure 5.4 *Fold*.
15. Secondary structure prediction using 2D DMS M^2^ data with RNAstructure 5.6 *Fold*.
16. Secondary structure prediction using 2D DMS M^2^ data with RNAstructure 5.6 *ShapeKnots*.

**Figure S2. Chemical mapping data and secondary structure predictions of RNA Puzzle 6.**

1. Secondary structure from crystallographic structures.
2. Secondary structure prediction without experimental data with RNAstructure 5.4 or 5.6 *Fold*. Nucleotides are colored with SHAPE reactivities. Crystallographic pairings missing in this model and new non-crystallographic pairings are drawn as yellow and blue lines, respectively. Percentage labels give bootstrap support values.
3. Secondary structure prediction using no data with RNAstructure 5.6 *ShapeKnots*.
4. Secondary structure prediction using 1D SHAPE data with RNAstructure 5.6 *Fold*.
5. Secondary structure prediction using 1D SHAPE data with RNAstructure 5.6 *ShapeKnots*.
6. Secondary structure prediction using 1D DMS/CMCT data with RNAstructure 5.4 *Fold*.
7. Secondary structure prediction using 1D DMS/CMCT data with RNAstructure 5.6 *Fold*.
8. Secondary structure prediction using 1D DMS/CMCT data with RNAstructure 5.6 *ShapeKnots*.
9. Secondary structure prediction using 1D SHAPE and DMS/CMCT data with RNAstructure 5.6 *Fold*.
10. Secondary structure prediction using 1D SHAPE and DMS/CMCT data with RNAstructure 5.6 *ShapeKnots*.
11. Secondary structure prediction using 2D SHAPE M^2^ data with RNAstructure 5.6 *Fold*.
12. Secondary structure prediction using 2D SHAPE M^2^ data with RNAstructure 5.6 *ShapeKnots*.

**Figure S3. Chemical mapping data and secondary structure predictions of RNA Puzzle 10.**

1. Secondary structure from crystallographic structures.
2. Secondary structure prediction without experimental data with RNAstructure 5.4 or 5.6 *Fold*. Nucleotides are colored with SHAPE reactivities. Crystallographic pairings missing in this model and new non-crystallographic pairings are drawn as yellow and blue lines, respectively. Percentage labels give bootstrap support values.
3. Secondary structure prediction using no data with RNAstructure 5.6 *ShapeKnots*.
4. Secondary structure prediction using 1D SHAPE data with RNAstructure 5.6 *Fold*.
5. Secondary structure prediction using 1D SHAPE data with RNAstructure 5.6 *ShapeKnots*.
6. Secondary structure prediction using 1D DMS/CMCT data with RNAstructure 5.4 *Fold*.
7. Secondary structure prediction using 1D DMS/CMCT data with RNAstructure 5.6 *Fold*.
8. Secondary structure prediction using 1D DMS/CMCT data with RNAstructure 5.6 *ShapeKnots*.
9. Secondary structure prediction using 1D SHAPE and DMS/CMCT data with RNAstructure 5.6 *Fold*.
10. Secondary structure prediction using 1D SHAPE and DMS/CMCT data with RNAstructure 5.6 *ShapeKnots*.

**Figure S4. Chemical mapping data and secondary structure predictions of RNA Puzzle 5.**

1. Normalized reactivity of RNA Puzzle 5 RNA, using SHAPE (1M7), DMS and CMCT in 1-dimensional chemical mapping. Reactivities were normalized to GAGUA referencing hairpins (not shown).
2. Secondary structure prediction using 1-dimensional SHAPE (1M7) data. Nucleotides are colored with SHAPE reactivities. Crystallographic pairings missing in this model and new non-crystallographic pairings are drawn as yellow and blue lines, respectively. Percentage labels give bootstrap support values.
3. Mutate-and-map (M^2^) dataset probed by the SHAPE reagent 1M7.
4. Secondary structure prediction using 2D SHAPE M^2^ data.

**Figure S5. Chemical mapping data and secondary structure predictions of RNA Puzzle 6.**

1. Normalized reactivity of RNA Puzzle 6 RNA, using SHAPE (1M7), DMS and CMCT in 1-dimensional chemical mapping, in presence of 60 μM adenosylcobalamin. Reactivities were normalized to GAGUA referencing hairpins (not shown).
2. Secondary structure prediction using 1-dimensional SHAPE (1M7) data. Nucleotides are colored with SHAPE reactivities. Crystallographic pairings missing in this model and new non-crystallographic pairings are drawn as yellow and blue lines, respectively. Percentage labels give bootstrap support values.
3. Mutate-and-map (M^2^) dataset probed by the SHAPE reagent 1M7, in presence of 60 μM adenosylcobalamin.
4. Secondary structure prediction using 2D SHAPE M^2^ data.

**Figure S6. Chemical mapping data and secondary structure predictions of RNA Puzzle 10.**

1. Normalized reactivity of RNA Puzzle 10 RNA, using SHAPE (1M7), DMS and CMCT in 1-dimensional chemical mapping, in presence of 1 μM partner RNA strand. Reactivities were normalized to GAGUA referencing hairpins (not shown).
2. Secondary structure prediction using 1-dimensional SHAPE (1M7) data. Nucleotides are colored with SHAPE reactivities. Crystallographic pairings missing in this model and new non-crystallographic pairings are drawn as yellow and blue lines, respectively. Percentage labels give bootstrap support values.
